# Downregulation of *NEAT1* due to loss of TDP-43 function exacerbates motor neuron degeneration in amyotrophic lateral sclerosis

**DOI:** 10.1093/braincomms/fcaf261

**Published:** 2025-07-02

**Authors:** Yu Kawakami, Yohei Iguchi, Jiayi Li, Yoshinobu Amakusa, Takashi Yoshimura, Ryo Chikuchi, Satoshi Yokoi, Madoka Iida, Yuichi Riku, Yasushi Iwasaki, Tetsuro Hirose, Shinichi Nakagawa, Masahisa Katsuno

**Affiliations:** Department of Neurology, Nagoya University Graduate School of Medicine, 65 Tsurumai-cho, Showa-ku, Nagoya, Aichi 466-8550, Japan; Department of Neurology, Nagoya University Graduate School of Medicine, 65 Tsurumai-cho, Showa-ku, Nagoya, Aichi 466-8550, Japan; Department of Neurology, Nagoya University Graduate School of Medicine, 65 Tsurumai-cho, Showa-ku, Nagoya, Aichi 466-8550, Japan; Department of Neurology, Nagoya University Graduate School of Medicine, 65 Tsurumai-cho, Showa-ku, Nagoya, Aichi 466-8550, Japan; Department of Neurology, Nagoya University Graduate School of Medicine, 65 Tsurumai-cho, Showa-ku, Nagoya, Aichi 466-8550, Japan; Department of Neurology, Nagoya University Graduate School of Medicine, 65 Tsurumai-cho, Showa-ku, Nagoya, Aichi 466-8550, Japan; Department of Neurology, Nagoya University Graduate School of Medicine, 65 Tsurumai-cho, Showa-ku, Nagoya, Aichi 466-8550, Japan; Department of Pathophysiological Laboratory Sciences, Nagoya University Graduate School of Medicine, Nagoya, Aichi 466-8550, Japan; Department of Neurology, Nagoya University Graduate School of Medicine, 65 Tsurumai-cho, Showa-ku, Nagoya, Aichi 466-8550, Japan; Department of Neurology, Nagoya University Graduate School of Medicine, 65 Tsurumai-cho, Showa-ku, Nagoya, Aichi 466-8550, Japan; Institute for Medical Science of Aging, Aichi Medical University, Nagakute, Aichi 480-1195, Japan; Institute for Medical Science of Aging, Aichi Medical University, Nagakute, Aichi 480-1195, Japan; Graduate School of Frontier Biosciences, Osaka University, Yamadaoka, Suita 565-0871, Japan; Institute for Open and Transdisciplinary Research Initiatives, Osaka University, Yamadaoka, Suita 565-0871, Japan; Faculty of Pharmaceutical Sciences, Hokkaido University, Sapporo 060-0808, Japan; Department of Neurology, Nagoya University Graduate School of Medicine, 65 Tsurumai-cho, Showa-ku, Nagoya, Aichi 466-8550, Japan; Department of Clinical Research Education, Nagoya University Graduate School of Medicine, Nagoya, Aichi 466-8550, Japan

**Keywords:** amyotrophic lateral sclerosis, TDP-43, *NEAT1*, mouse model, neurodegeneration

## Abstract

TAR DNA-binding protein 43 (TDP-43) is of particular interest in the pathogenesis of amyotrophic lateral sclerosis (ALS). It has been speculated that loss of nuclear TDP-43 and its cytoplasmic aggregation contributes to neurodegeneration. Although considerable attention has been paid to RNA metabolism in TDP-43 function, TDP-43 is also known to act as a transcription factor. This study found that the expression of Nuclear-enriched abundant transcript 1 (*NEAT1*), a long-non-coding RNA, was substantially downregulated in motor neurons with nuclear TDP-43 loss, but upregulated in those with preserved nuclear TDP-43, in the postmortem spinal cords of patients with sporadic ALS. TDP-43 depletion induced *Neat1* downregulation in Neuro2a cells, primary cortical neurons, and mouse spinal motor neurons. Furthermore, TDP-43 was found to positively regulate *NEAT1* at the transcriptional level. Finally, *Neat1* knockout exacerbates neurodegeneration of hSOD1^G93A^ mice accompanied by increased misfolded superoxide dismutase 1 (SOD1) aggregations. Transcriptome analysis revealed that *Neat1* knockout reduced protein folding-related genes, such as heat shock protein family A member 1A (*Hspa1a*), in the spinal cords of hSOD1^G93A^ mice. Our results indicated that the loss of TDP-43 function enhances ALS neurodegeneration by losing the protective effect of *NEAT1*.

## Teaser

Kawakami *et al*. reported that the expression of long non-coding RNA nuclear-enriched abundant transcript 1 (*NEAT1*) is downregulated in motor neuron with loss of nuclear TAR DNA-binding protein (TDP-43) in amyotrophic lateral sclerosis. *In vivo* experiment revealed that *NEAT1* depletion exacerbates motor neuron degeneration with downregulation of protein folding-related gene.

## Graphical Abstract

**Graphical Abstract:**
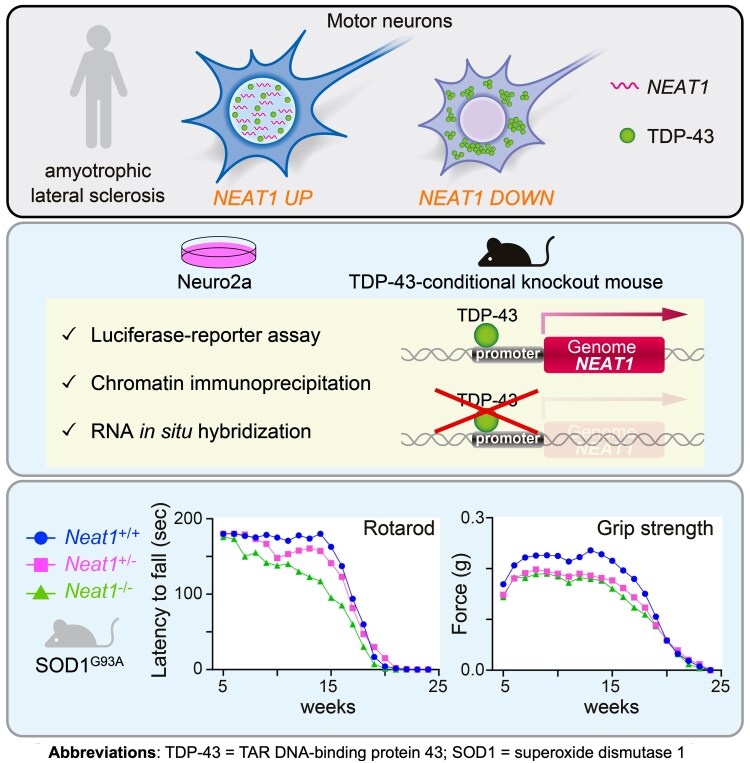

## Introduction

Amyotrophic lateral sclerosis (ALS) is a progressive and fatal neurodegenerative disease that affects the upper and lower motor neurons. Approximately 90% of ALS cases are sporadic; however, its pathogenesis remains unknown.^1,2^ TAR DNA-binding protein 43 (TDP-43) was identified as a major component of the ubiquitinated cytoplasmic inclusion bodies in ALS motor neurons.^3,4^ Although TDP-43 is a nuclear protein, it disappears from the nucleus in degenerating ALS neurons. Thus, it has been speculated that the pathogenicity associated with TDP-43 involves loss of function and exacerbates toxicity owing to cytoplasmic aggregates.^5^ Mice with forced expression of cytoplasmic TDP-43 showed motility defects.^6^ Furthermore, it was reported that crossing normal human TDP-43-expressing mice with Q331K-expressing mice promoted neuronal degeneration, with nuclear TDP-43 loss and intracellular aggregation in motor neurons.^7^ These data support a gain of toxicity caused by TDP-43 cytoplasmic aggregation. Meanwhile, studies on TDP-43 function loss have been conducted on many organisms, including cultured neurons,^8^ conditional TDP-43 knockout mice,^9^  *Drosophila*,^10^ and zebrafish.^11^ TDP-43 depletion has been recently reported to promote the cryptic exon of several genes, such as stathmin-2 and UNC13A, which downregulate their transcriptions.^12-15^ As these molecules are indispensable for neuronal activity, the loss of TDP-43 function has increasingly gained attention as a cause of ALS pathology. Although considerable attention has been paid to RNA metabolism in TDP-43 function, TDP-43 is also known to act as a transcription factor.^16-18^

Nuclear-enriched abundant transcript 1 (*NEAT1*) is a long-non-coding RNA that forms two isoforms, *NEAT1_1* (3.7 kb) and *NEAT1_2* (23 kb).^19,20^  *NEAT1_2* is an essential component of the paraspeckle skeleton.^21,22^  *NEAT1_2* expression is upregulated in the early stages of motor neuron pathology in ALS and then gradually decreases as the disease progresses.^23^ The upregulation of *NEAT1* in ALS is speculated to be a protective response to enhance cellular stress resistance.^24,25^ However, the mechanism regulating *NEAT1* expression and the effect of *NEAT1* expression change in ALS motor neurons remain unclear.

This study found that nuclear TDP-43 loss is well correlated with *NEAT1* depletion in ALS motor neurons and that TDP-43 regulates *NEAT1* at the transcriptional level. *Neat1* knockout exacerbates the neurodegeneration of G93A mutant form of human superoxide dismutase 1 transgenic mice (hSOD1^G93A^ mice) with a reduction of genes associated with protein folding.

## Materials and methods

### Sex as a biological variable

Our study examined male and female animals, and similar findings are reported for both sexes.

### Human tissue

Post-mortem human spinal cord tissue specimens were obtained by autopsy with informed consent. Patients’ ALS diagnosis was based on the Awaji criteria, the revised El Escorial criteria, and histopathological confirmation of the presence of TDP-43 pathology. Anonymized clinical patient information is as follows: control 1, dementia with Lewy bodies, 82 y/o, female; control 2, multiple system atrophy with predominant cerebellar ataxia, 75 y/o, male; control 3, familial amyloid polyneuropathy, 71 y/o, male; control 4, polyarteritis nodosa, 76 y/o, female; control 5, tuberculosis, 55 y/o, male; control 6, cerebral infarction, 86 y/o, female; control 7, duodenal ulcer, 68 y/o, male; control 8, gastric ulcer, 76 y/o, male; ALS 1, 68 y/o, female; ALS 2, 85 y/o, male; ALS 3, 68 y/o, male; ALS 4, 61 y/o, male; ALS 5, 86 y/o, female; ALS 6, 65 y/o, male; ALS 7, 87 y/o, female; ALS 8, 70 y/o, male. This study was conducted in accordance with the Ethical Guidelines for Human Genome/Gene Analysis Research and Ethical Guidelines for Medical and Health Sciences Research Involving Human Subjects of Japan. The use of human biological materials was approved by the Ethics Review Committee of Nagoya University.

### RNA-FISH with immunofluorescence for human and mouse sample

Paraffin sections (6 µm) of human spinal cord from ALS patients and controls were used for FISH of *NEAT1*-total, *NEAT1_2*, or *NEAT1_2* negative control and immunofluorescent double staining of TDP-43, non-POU domain containing octamer binding (NONO), or splicing factor proline and glutamine rich (SFPQ). Paraffin section (3 µm) of mouse spinal cord from TDP-43-cKO mice and controls were used for FISH of *Neat1*-total, *Neat1_2*, or Choline acetyltransferase (*Chat*) and immunofluorescent double staining of TDP-43. RNA-FISH was performed using the RNAscope TM Multiplex Fluorescent Reagent Kit v2 (323100; Advanced Cell Diagnostic) and was performed according to the manufacturer's instructions. Then sections were encapsulated with ProLong Gold Antifade Reagent with 4′,6-diamidino-2-phenylindole (DAPI) (P36935; Thermo Fisher Scientific). Probes and antibodies used were for human tissue are as follows: RNAscope probe-Hs-NEAT1-short (411531; Advanced Cell Diagnostic), RNAscope probe-Hs-NEAT1-long (411541), and RNAscope probe-Hs-NEAT1-long-scramble (553701); and NONO mouse monoclonal (611279; 1:1000; BD Biosciences), SFPQ mouse monoclonal (RN014MW; 1:1000; MBL). For mouse tissue, RNAscope probe-Mm-Neat1-C2 (440351-C2), RNAscope probe-Mm-Neat1-01 (514921), RNAscope probe-Mm-Chat (408731) and RNAscope probe-Mm-Chat-C2 (408731-C2); and TDP-43 rabbit polyclonal (10782-2; 1:1000; Proteintech). Confocal images were taken using LSM 880-ELYRA PS.1 (Carl Zeiss) confocal microscope. Especially in human spinal motor neurons, lipofuscin, a granular lipophilic pigment which accumulates with age, is often detected with autofluorescence intensities between 500 and 695 nm.

### Antibodies

For immunofluorescent and immunohistochemistry, following antibodies were used: TDP-43 rabbit polyclonal (10782-2; 1:1000; Proteintech); TDP-43 (c-terminal) rabbit polyclonal (12892-1-AP; 1:1000; Proteintech); V5 chicken polyclonal (A190-118A; 1:1000; Bethyl Laboratories); TDP-43 pSer409 rabbit polyclonal (TIP-PTD-P03; 1:1000; Cosom Bio); Ubiquitin mouse monoclonal (sc-166553; 1:1000; Santa Cruz Biotechnology); ChAT goat polyclonal (AB144P; 1:1000; MilliporeSigma); Neurofilament-L (NfL) rabbit polyclonal (#2837; 1:200; Cell Signalling); synaptic vesicle protein 2 (SV2) mouse monoclonal (SV2-c; 1:200; DSHB); Cleaved caspase 3 rabbit polyclonal (9661L; 1:500; Cell Signalling); HSPA1A rabbit polyclonal (PA5-34772 1:500; Thermo Fisher Scientific); and Misfolded SOD1 mouse monoclonal (B8H10) (MM-0070-P; 1:50; MEDIMABS). For immunoblotting, following antibodies were used: TDP-43 rabbit polyclonal (10782-2; 1:1000; Proteintech); TDP-43 (C-terminal) rabbit polyclonal (12892-1-AP; 1:1000; Proteintech); Glyceraldehyde-3-phosphate dehydrogenase (GAPDH) mouse monoclonal (ab8245; 1:5000; Abcom); FLAG rabbit polyclonal (15009; 1:1000; Cell Signalling); HSPA1A rabbit polyclonal (PA5-34772 1:500; Thermo Fisher Scientific); and Misfolded SOD1 mouse monoclonal (B8H10) (MM-0070-P; 1:50; MEDIMABS).

### DNA constructs

GKPIPNPLLGLDST (V5)-tagged human TDP-43-wild type (WT), V5-TDP-43-modified NLS (mNLS), and V5-TDP-43-modified NLS and RRM (mNmR) plasmid were created as previously described.^26-28^ NLS and RRM stand for nuclear localisation signal and RNA recognition motif, respectively. To generate a DYKDDDDK (FLAG)-tagged-mouse TDP-43 plasmid, pEX-A2J1 plasmid with mouse TDP-43 cDNA (Eurofins) was purchased and TDP-43 cDNA was inserted into the pENTR4-FALG (w210-2; addgene) with restrict enzymes. Then, FLAG-TDP-43 was inserted into pLenti-CMV/TO-Puro DEST (670-1; addgene) using Gateway LR Clonas II Enzyme mix (11791020; Thermo Fisher Scientific). FLAG-tagged TDP-43 with modified RRM1 (FLAG-TDP-43-mRRM) was produced by mutagenesis with each pair of the primers mTDP-43-C175S-F; 5′-GACTCTAAACTTCCCAACTCTAAGC-3′ and mTDP-43-C173S-R; 5′-AGACCATCGCCCATCTATC-3′. The siRNA-resistant form of the TDP-43 gene (FLAG-siRNA-resistant TDP-43 and FLAG-siRNA-resistant TDP-43-mRRM) was generated by changing the targeted sequence of the siRNA to 5′-AAATGACGAGCCAATAGA-3′ (mutated nucleotides are underlined) with pair of the primers synonymous-mTDP-F1; 5′-GCCAATAGAAATACCATCAGAAGACGATGGG-3′ and -R1; 5′-TCGTCATTTTCATCTTCTGTTACCCGAATATATT-3′ using the KOD-Plus-Mutagenesis kit (Toyobo, Osaka, Japan).

### Cell cultures and transfection

The Neuro2a cell line was cultured in Dulbecco's Modified Eagle's medium supplemented with 10% fetal bovine serum in an incubator at 37°C in an atmosphere of 95% air/5% CO_2_. All cell lines were obtained from ATCC and tested negative for mycoplasma contamination. For transfection of siRNA into Neuro2a cells was performed with Lipofectamine RNAiMAX (13778-075; Thermo Fisher Scientific) according to the manufacturer's instructions. For the transfection of plasmid and siRNA, cells were co-transfected using Lipofectamine 3000 Reagent (L3000-015; Thermo Fisher Scientific) and RNAiMAX, respectively. For intervention experiments, cells were harvested 48 h after transfection. Primary cortical neurons were prepared from TDP-43^flox/flox^ mouse^9^ embryos of E15 as previously described.^29^ The primary neurons were cultured in neurobasal medium (Gibco) supplemented with B27 (Gibco) and GluMax (Gibco). At DIV1, cells were transduced with EGFP-Cre or EGFP lentivirus. At DIV9, the cells were used for Western blotting and qRT-PCR.

### Lentivirus production

Lentiviruses were prepared as previously described.^29^ About 24 h before transfection, 293T cells were passaged in DMEM adding 10% FBS. The cells were transfected with 3-μg pLV-EGFP-Cre (86805; Addgene) or pLV-EGFP (w159-1) (17481; Addgene) vector, 2-μg pLP1, 2-μg pLP2, 1-μg pVSVG, and Lipofectamine 2000 in OptiMEM. Three hours after transfection, the medium was replaced with fresh DMEM adding 10% FBS. Two days after incubation, the medium was filtered and stored at −80°C.

### Neuro2a immunofluorescence

The transfected Neuro2a cells grown on glass slides were fixed in 4% paraformaldehyde buffer for 30 min and incubated with 1% Triton X-100 (9036-19-5; Sigma-Aldrich) and PBS for 5 min. The cells were blocked with Tris-NaCl blocking buffer (FP1012; PerkinElmer) for 1 and then incubated with first antibodies [anti-V5, anti-ubiquitin, anti-phosphorylated TDP-43, or anti-TDP-43 (c-terminal)] overnight at 4°C. The next day, the cells were incubated with secondary antibodies for 1 h and then encapsulated with ProLong Gold Antifade Reagent with DAPI (P36935; Thermo Fisher Scientific). Confocal images were acquired with a confocal system (LSM 880-ELYRA PS.1; Carl Zeiss). Images were captured using ZEN (black edition) 2.3 software. The following antibodies were used as secondary antibodies: Alexa Fluor 488 goat anti-chicken IgG (A11039; 1:000; Thermo Fisher Scientific), Alexa Fluor 568 goat anti-rabbit IgG (A11036; 1:000; Thermo Fisher Scientific), and Alexa Fluor 633 goat anti-mouse IgG (A21052; 1:000; Thermo Fisher Scientific).

### Quantitative RT-PCR

Quantitative RT-PCR was used to evaluate the *Neat1*-total, *Neat1_2, Hspa1a*, heat shock protein family A member 1B *(Hspa1b)*, and heat shock protein family A member 2 *(Hspa2)* expression. The RNeasy Mini Kit (74104; QIAGEN) was used to isolate total RNA from Neuro2a cells, mouse primary cortical neurons, and cervical spinal cords of *Neat1*-KO; hSOD1^G93A^ and *Neat1-*WT; hSOD1^G93A^ mice at 8 weeks according to the manufacturer's instructions. Prior to total RNA extraction, cell or tissue lysates in QIAzol Lysis reagent (108-95-2; QIAGEN) were passed 20 times through a 26-gauge needle or heated 55°C for 10 min with about 1400 rpm agitation (Micro tube mixer MT-400; TOMY). After RNA extraction, cDNA synthesis was performed by SuperScript VILO cDNA Synthesis Kit (11755050; Thermo Fisher Scientific) and used for quantitative real-time PCR using Thunderbird SYBR qPCR Mix (QPS-201; TOYOBO) and iCycler detection system (170-8740; Bio-Rad). Expression levels were normalized to Beta-2 microglobulin (β2MG). The following primers were used: mouse *Neat1*-total: 5′-TGGAGATTGAAGGCGCAAGT-3′ and 5′-ACCACAGAAGAGGAAGCACG-3′; mouse *Neat1_2*: 5′-GAAGACTCCTGCTGTGACCA-3′ and 5′-TTGGGCTAACTCAAGTGCAG-3′; mouse *Hspa1a*: 5′-TGGTGCAGTCCGACATGAAG-3′ and 5′-GCTGAGAGTCGTTGAAGTAGGC-3′; mouse *Hspa1b*: 5′-GAGATCGACTCTCTGTTCGAGG-3′ and 5′-GCCCGTTGAAGAAGTCCTG-3′; mouse *Hspa2*: 5′-GCGTGGGGGTATTCCAACAT-3′ and 5′-TGAGACGCTCGGTGTCAGT-3′; mouse β2MG: 5′-CTGACC GGCCTGTATGCTAT-3′ and 5′-CCGTTCTTCAGCATTTGGAT-3′.

### siRNAs

The oligonucleotide siRNA duplex was synthesized by Takara Bio (Sigma, Japan). The siRNA-sequences were as follows: scrambled (control) siRNA, 5′-GAAUCAGAUGCACAUGAGUUU-3′; TDP-43 siRNA-#1, 5′-GAACGAUGAACCCAUUGAAUU-3′; -#2, 5′-GUUCUUAUGGUUCAGGUCAUU-3′. Unless otherwise mentioned, #1 siRNA was used for TDP-43 knockdown throughout the experiments.

### Western blotting

For the analysis of Neuro2a cells lysates and mouse primary neurons, the cells and mouse primary cortical neurons were collected with 200 µL RIPA buffer (50 mM Tris-HCl pH 7.4, 150 mM NaCl, 1% Triton X-100, 1% Sodium Deoxycholate, and 0.1% SDS) with Half Protease and Phosphatase inhibitor cocktail (1861281; 1:100; Thermo Fisher Scientific). Following sonication and denaturation at 95°C for 10 min, samples were lysed in 3× sample buffer. For analysis of protein solubility analysis, mouse spinal cords were lysed in 300 µL lysis buffer (25 mM Tris-HCl pH 7.4, 150 mM NaCl, 1 mM EDTA, 1% NP-40, and 5% glycerol) with Halt Protease and Phosphatase inhibitor cocktail (1861281; 1:100; Thermo Fisher Scientific). Following sonication and centrifugation at 15 000 × g for 10 min, the supernatant was collected as soluble samples, and the pellets were lysed in 200 µL 1× sample buffer as insoluble samples. Samples were boiled for 10 min. Sample lysates were separated by SDS-PAGE and analyzed by Western blotting with ECL Prime Western Blotting System (GERPN2232; Cytiva). Primary antibodies used were previously described and secondary antibodies were anti-mouse IgG-HRP (NA9310V; 1:5000; Cytiva) and anti-rabbit IgG-HRP (458; 1:5000; MBL). Protein expression was quantified with LAS-3000 Luminescent Image Analyzer (GE Healthcare Life Sciences) and FUSION SOLO7S (Vilber-Lourmat).

### RNA-FISH for Neuro2a

Neuro2a cells transfected with control siRNA or siTDP-43 on glass slides were used for FISH of *Neat1_2*. RNA-FISH was performed using the RNAscope TM Multiplex Fluorescent Reagent Kit v2 (323100; Advanced Cell Diagnostic) and was performed according to the manufacturer's instructions. Then cells were encapsulated with ProLong Gold Antifade Reagent with DAPI (P36935; Thermo Fisher Scientific). Probe of RNAscope probe-Mm-Neat1-01 (514921) were used. Confocal images were taken using LSM 880-ELYRA PS.1 (Carl Zeiss) confocal microscope.

### Motor neuron-specific conditional TDP-43 knockout mice

Motor neuron-specific TDP-43 cKO (TDP-43^flox/flox^/VAChT-Cre) mice were produced as previously described.^9^ The TDP-43^flox/flox^ mice were used as control.

### Luciferase reporter assay

A *NEAT1*-promoter luciferase vector^30^ is a reporter plasmid containing the luciferase gene under the control of the human *NEAT1* promoter (−951 to +110). Neuro2a cells seeded into 96-well plates were transfected with the *NEAT1*-promoter luciferase vector or pGL3-control vector (Promega) together with control siRNA or two types of siTDP-43. Simultaneously, pNL1.1.PGK_Nluc plasmid (Promega) was co-transfected to monitor the number of culture cells per well. At 48 h after the transfection, luciferase assays were performed using the Nano-Glo Dual-Luciferase Reporter Assay System (N1610; Promega) according to the manufacturer's instructions.

### Chromatin immuno precipitation assay

Neuro2a cells seeded into 10 cm dishes were transfected with pLV-FLAG-mock, pLV-FLAG-TDP-43, or pLV-FALG-TDP-43-mRRM. At 24 h after transfection, chromatin immuno precipitation assay (ChIP) assays were performed with anti-FALG antibody (66008-4-Ig; Proteintech) or normal mouse Immunoglobulin G (IgG) as a control using a ChIP Assay Kit (#26157; Thermo Scientific) according to the manufacturer's instructions. The ChIP DNAs are subjected to quantitative PCR. The primer sequences for *NEAT1*-promoter region are 5′-GCGCTCTTCAACCATAAACA-3′ and 5′-GCTTGGGTGGAATGCTTAAT-3′.

### Animals

All mice were housed in clear plastic cages with free access to water and food under a temperature of 25°C, humidity of 55% ± 10%, and a 12-h light/dark cycle. By crossing *Neat1^+/−^*; hSOD1^G93A^ male mice with *Neat1^+/−^* female mice, we generated each genotype: *Neat1*^+/+^ (*n* = 12, Male/Female = 6/6), *Neat1*^+/−^ (*n* = 14, Male/Female = 7/7), *Neat1*^−/−^ (*n* = 15, Male/Female = 7/8), *Neat1^+/+^*; hSOD1^G93A^ (*n* = 21, Male/Female = 12/9), *Neat1^+/−^*; hSOD1^G93A^ (*n* = 22, Male/Female = 11/11), *Neat1^−/−^*; hSOD1^G93A^ (*n* = 19, Male/Female = 11/9). Generation of the *Neat1* knockout (*Neat1*-KO) mouse strain has been described previously.^31^ The sample size was decided with reference to previous report.^32^ Mice transgenic for human SOD1^G93A^ were purchased from the Jackson Laboratory (Bar Harbor, ME) and maintained as hemizygotes by mating transgenic males with B6/ SJLF1 females.^33^ Male and female mice were used for all experiments and experimental groups were sex balanced. Body weight was recorded every week throughout disease progression. Disease onset was defined as the last 180-s week of the rotarod test. Disease endstage was determined when either loss of righting reflex for more than 15 s or loss of 30% of body weight, whichever was observed soonest. No individuals were excluded. All mice were housed and maintained in accordance with the Guide for the Care and Use of Laboratory Animals of the National Institutes of Health and approved by the Institutional Committee Under Regulations on Animal Experiments of Nagoya University.

### Behavioural assessment

Motor balance, muscle strength, and coordination of *Neat1*-KO; hSOD1^G93A^ mice were measured using a Rotarod 47600 with 5 Lanes (UGO BASILE). The speed was set to 16 rpm, and recording was terminated when the number of seconds from measurement to fall or 180 s had elapsed. 4-week-old mice were trained for 2 days. Rotarods were performed once a week from 5 to 24 weeks of age. Limb grip strength was measured using a MK-380M grip strength metre (Muromachi Kikai). The maximum value obtained in three consecutive trials was used as the record; measurements were taken once a week from 5 to 24 weeks of age. Rotarod and grip strength measurements were evaluated on the same day by researcher blinded to genotype.

### Mouse neuromuscular junction analysis

Anterior tibialis muscles were harvested from 8- and 16-week-old mice, fixed in 4% paraformaldehyde for 1 h, then placed in PBS and stored at 4°C. Muscle fibres were teased directly on slides to obtain individual fibres. The muscle fibres were treated in 4% Tritonx-100/PBS at room temperature for 90 min and then with PBS including 4% BSA and 2% Triton X-100 as blocking buffer at room temperature for 30min. Muscle sections were stained with a cocktail of anti-SV2 and anti-Neurofilament L to fully label both the axon and the nerve terminal and biotin conjugates of alpha bungarotoxin (B1196; 1:1000; Thermo Fisher Scientific) to label postsynaptic receptors. Images were acquired with a confocal microscope (LSM 880-ELYRA PS.1; Carl Zeiss) using a Z-series. NMJs were considered innervated if at least 50% of a-bungarotoxin (BTX)-labelled motor endplates overlapped with SV2-labelled nerve terminal. For each individual, about 70–110 NMJs were observed and the innervated NMJs were counted manually.

### Mouse motor neuron analysis

Lumbar spinal cords from 4-week-old, 8-week-old, 16-week-old, and endstage mice were used for immunofluorescence. Images of spinal cord sections taken by confocal microscope (LSM 880-ELYRA PS.1; Carl Zeiss) were analyzed manually. ChAT-positive neurons in the ventral horn of the spinal cord were counted in planar images with the nucleolus most clearly visible. The ChAT-positive neurons in the ventral horns were regarded as motor neurons. In addition, the ratio of cleaved caspase 3 positivity cells per ChAT-positive motoneuron was calculated.

### Transcriptome analysis RNA-sequencing

RNAs isolated from cervical spinal cords of *Neat1*-KO; hSOD1^G93A^ (*n* = 4) and *Neat1-WT*; hSOD1^G93A^ (*n* = 3) mice at 8 weeks of age were submitted. RNA-seq analyses were performed by the pipeline provided by Rhelixa, Inc. Sequencing was performed using the Illumina NovaSeq 6000 Sequencing System. Libraries were prepared using the NEBNext Poly(A) mRNA Magnetic Isolation Module (E7490; NEB) and NEBNext Ultra II Directional RNA Library Prep Kit for Illumina (E7530). The quality of the raw paired-end sequence reads was assessed with FastQC (Version 0.11.7; https://www.bioinformatics.babraham.ac.uk/projects/fastqc/). Low quality (<20) bases and adapter sequences were trimmed using Trimmomatic software (Version 0.38) with the following parameters: ILLUMINACLIP: path/to/adapter.fa:2:30:10 LEADING:20 TRAILING:20 SLIDINGWINDOW:4:15 MINLEN:36. The trimmed reads were aligned to the reference genome by RNA-seq aligner HISAT2 (Version 2.1.0). The .sam files from the HISAT2 results were converted to .bam files with Samtools (Version 1.9). The .bam files were used to estimate the abundance of uniquely mapped reads using featureCounts (Version 1.6.3). Using the raw read counts, GO term and pathway enrichment analysis of differentially expressed genes were analyzed using iDEP.96 online tool (http://bioinformatics.sdstate.edu/idep96/).

### Mouse immunofluorescence and immunohistochemistry

The mice were anesthetized with medetomidine (0.3 mg/kg), midazolam (4 mg/kg), and butorphanol (5 mg/kg) and then perfused with 20 mL cold 4% paraformaldehyde phosphate buffer solution through the left cardiac ventricle. The spinal cords were removed, postfixed in formalin, and embedded in paraffin. Tissue sections (3 µm) were deparaffinized and dehydrated with alcohol. Antigen retrieval was performed with 10 mM citrate buffer (pH6.0) heated in a microwave for 15 min. The primary antibody was then incubated overnight at 4°C after blocking with TNB Blocking Buffer in 2% BSA for 60 min at room temperature. The primary antibodies used were HSPA1A rabbit polyclonal and misfolded SOD1 mouse monoclonal (B8H10). For immunofluorescence, the sections were incubated with secondary antibodies at room temperature for 1 h. Images were acquired using LSM 880-ELYRA PS.1 (Carl Zeiss). For immunohistochemistry, after reaction to primary antibodies, the sections were incubated with the EnVision + HRP System (Dako). Images were acquired using an Olympus BX51 microscope.

### Statistics

The statistical details of each experiment are described in the figure legends. All data were expressed as mean ± SEM. Two-tailed unpaired Student's *t*-test was used for the comparison between two groups. One-way analysis of variance (ANOVA) with Tukey's multiple comparisons test was used to compare three or more groups. Two-way ANOVA with Tukey's multiple comparisons test was used for the mouse behavioural experiments. All statistical data were analyzed using GraphPad Prism 9 (GraphPad Software, Inc., La Jolla, CA).

## Results

### *NEAT1* is reduced in the spinal motor neurons of sporadic ALS cases accompanied by TDP-43 pathology

**Figure 1:**
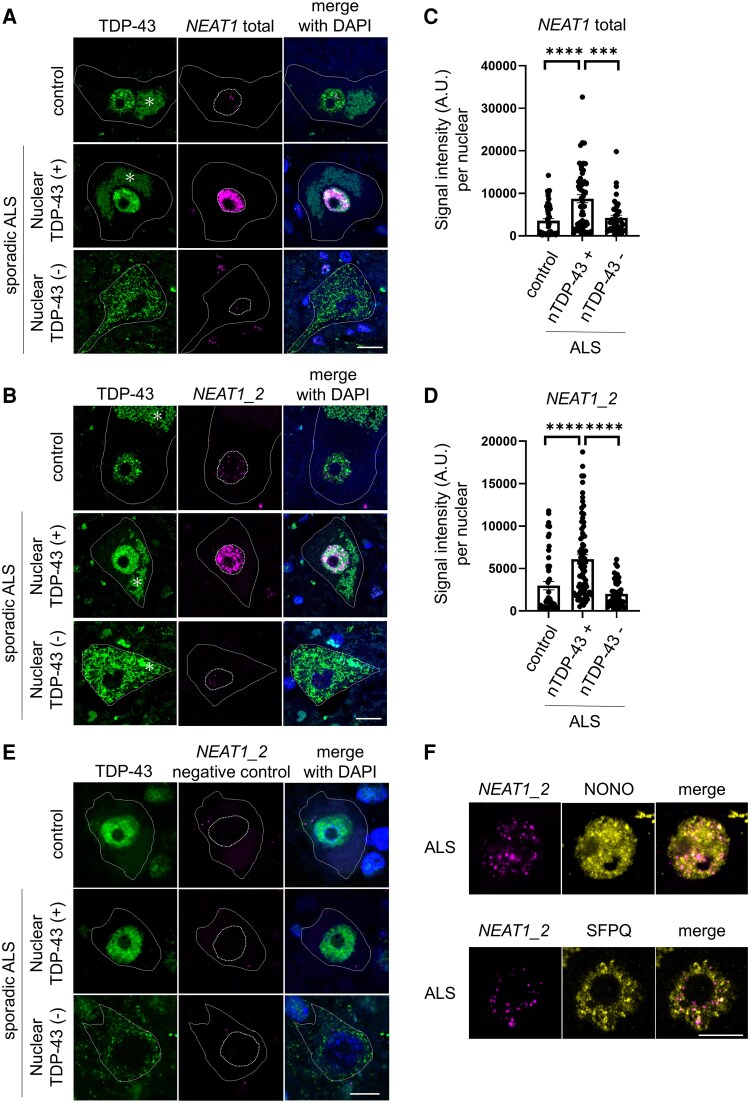
***NEAT1* expression with or without TDP-43 pathology in ALS spinal motor neurons**. (**A** and **B**) Postmortem spinal cords from patients with sporadic ALS and controls were thinned to 6 µm and stained via *in situ* hybridisation with *NEAT1*-total specific probe (**A**) or *NEAT1_2* specific probe (**B**) followed by double-immunostaining with anti-TDP-43 and DAPI to nuclear. The solid and dotted lines indicate the neuronal cell bodies and the nuclear, respectively. Scale bar: 10 µm. Asterisks: lipofuscin. (C and D) The signal intensities of *NEAT1*-total (**C**) and *NEAT1_2* (**D**) in spinal motoneurons in the control group and in groups with and without nuclear TDP-43 (nTDP-43+ and nTDP-43−, respectively) in ALS were measured. Each individual data point represents intensity of signal intensity per a nuclear for a single motor neuron. The data bars are expressed as mean ± SEM. Data were collected from 8 ALS patients and 8 controls. Statistical analysis was conducted via one-way ANOVA, followed by Tukey's multiple comparisons test. ***P < 0.001, ****P < 0.0001. (**E**) Postmortem spinal cords from patients with sporadic ALS and controls were stained via *in situ* hybridisation with *NEAT1_2* negative control probe (magenta) followed by double-immunostaining with anti-TDP-43 and DAPI to nuclear. (**F**) *In situ* hybridisation (*NEAT1_2*) and immunofluorescence images (NONO and SFPQ) of spinal motor neurons. Scale bar: 10 µm. A.U., arbitrary unit.

To evaluate the association between TDP-43 and *NEAT1* expression in ALS, we performed RNA fluorescence *in situ* hybridisation (FISH) and immunofluorescence double staining on the postmortem spinal cords of nine patients with sporadic ALS and eight control subjects (Fig. 1). The results indicated that the *NEAT1-total* expression was elevated in the ALS spinal motor neurons where TDP-43 remained in the nucleus compared with the control motor neurons but was reduced in the spinal motor neurons where TDP-43 was absent in the nucleus and aggregated in the cytoplasm (Fig. 1A and C). This pattern was also observed for the expression of *NEAT1_2*, which is a longer stress-inducible *NEAT1* isoform (Fig. 1B and D). We cannot specifically detect a shorter isoform, *NEAT1_1,* because *NEAT1_1* shares sequences with *NEAT1_2*. FISH with the *NEAT1_2* negative control probe did not exhibit any nuclear signal in any motor neurons (Fig. 1E), and most of the signals with the *NEAT1_2* probe colocalized with NONO and SFPQ, which are major components of the paraspeckle (Fig. 1F). While there is no significant difference in *NEAT1* expression in motor neurons with TDP-43 nuclear depletion in ALS compared with those in control samples, *NEAT1* is upregulated in ALS pathology, probably independently of TDP-43. Our results suggest that the reduction of *NEAT1* expression in ALS motor neurons is associated with the nuclear loss of TDP-43.

### Knockdown of TDP-43 reduces the expression level of *NEAT1* in the spinal motoneurons

We speculated that either TDP-43 loss or cytoplasmic TDP-43 aggregation promotes *NEAT1* reduction. To examine the effect of cytoplasmic TDP-43 aggregation, we created a TDP-43 construct with cytoplasmic localisation (TDP-43-mNLS) and a TDP-43 construct with a mutation causing pathological aggregation in the cytoplasm (TDP-43-mNmR)^26,28^ (Fig. S1A). In cultured Neuro2a cells, TDP-43-mNLS was diffusely expressed in the cytoplasm, whereas TDP-43-mNmR formed ubiquitin-positive aggregates in the cytoplasm, which were also positive for phosphorylated TDP-43 (Fig. S1B). Quantitative reverse transcription polymerase chain reaction (qRT–PCR) showed no significant changes in the expression levels of *Neat1*-total and *Neat1_2* (Fig. S1C and D). These results are consistent with previous report^34^ and suggest that neither cytoplasmic TDP-43 overexpression nor aggregation affects *NEAT1* expression.

**Figure 2:**
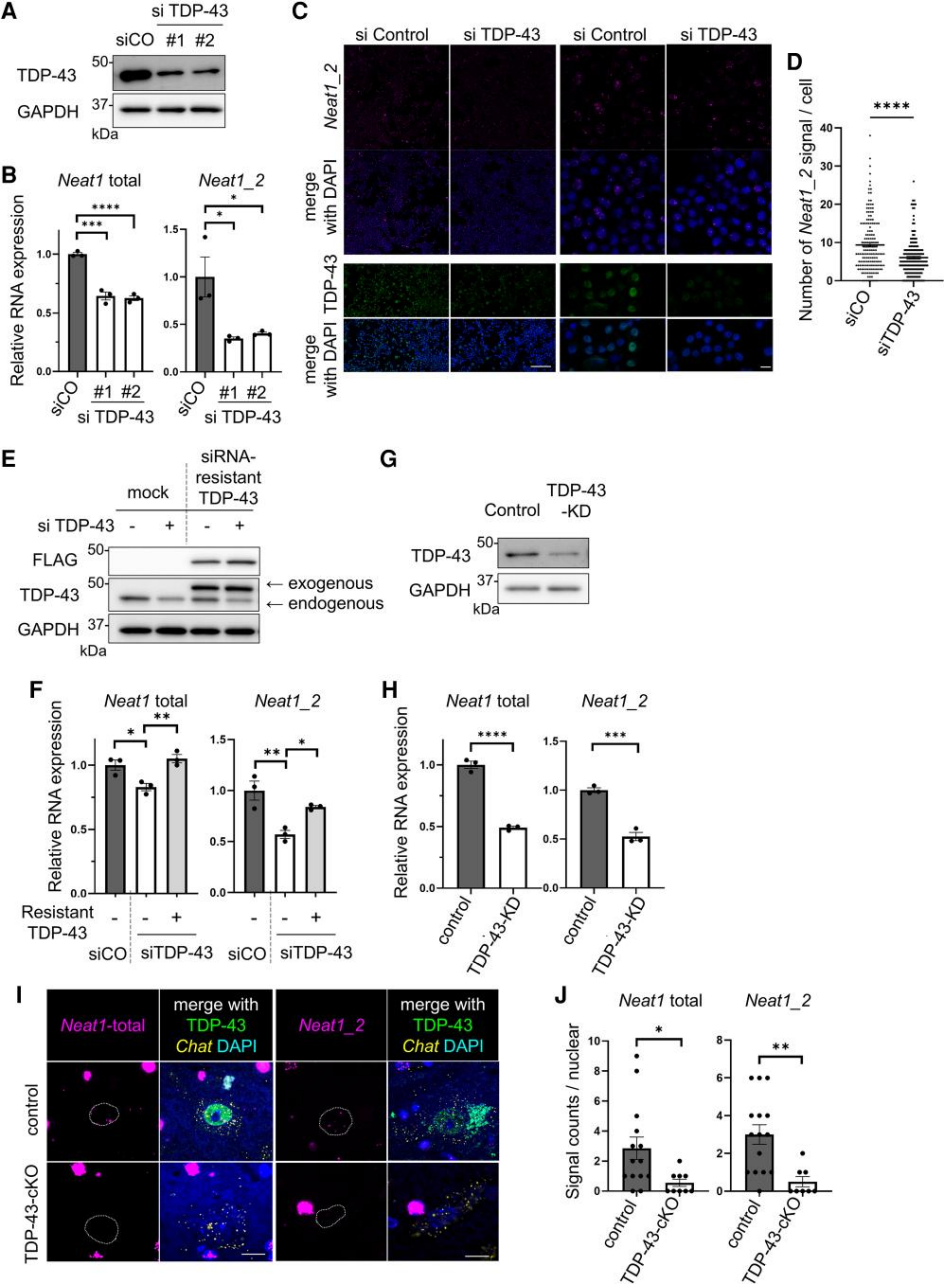
**Effect of TDP-43 depletion on *NEAT1* expression**. (**A**) Immunoblots of Neuro2a cell lysates treated with control siRNA (siCO) or each two siTDP-43 (#1 or #2). See supplementary Fig. 5 for uncropped blots. (**B**) Quantification of the mRNA expression levels of *Neat1*-total and *Neat1_2* using cells in A (*n* = 3 for each group). (**C**) Upper panels show in situ hybridisation (*Neat1_2*) of Neuro2 treated with siCO or siTDP-43, and under panels show immunostaining (TDP-43). Scale bar: Right = 10 µm, Left = 100 µm. (**D**) Number of *Neat1_2* signals per a Neuro2a cell (*n* = 162 for siCO group, *n* = 169 for siTDP-43 group). (**E**) Immunoblots of Neuro2a cell lysates expressing FLAG-siRNA-resistant-TDP-43 and control siRNA or siTDP-43. In the TDP-43 immunoblot lane, the upper and lower bands indicate transfected TDP-43 (FLAG-resistant) and endogenous TDP-43, respectively. See supplementary Fig. 6 for uncropped blots. (**F**) Quantification of the mRNA expression levels of *Neat1*-total and *Neat1_2* using cells in E (*n* = 3 for each group). (**G**) Immunoblots of whole tissue lysate derived from primary cortical neurons with TDP-43 knockdown by EGFP-Cre lentivirus. See supplementary Fig. 7 for uncropped blots. (**H**) Quantification of the mRNA expression levels of *Neat1*-total and *Neat1_2* using tissue lysate in G (*n* = 3 for each group). (**I**) *In situ* hybridisation (*Neat1*-total and *Neat1_2*) and immunofluorescence images (TDP-43, ChAT and DAPI) of spinal motoneurons in TDP-43-cKO mice. The dotted lines indicate the nuclear. Scale bar: 10 µm. (**J**) Signal counts of *Neat1*-total and *Neat1_2* in the nuclei of spinal motoneurons (*n* = 9–15 for each group). In B, F, and H, each individual data point represents the average of three measurement for a culture well. In D, each individual data point represents signal counts per a Neuro2a cell. In J, each individual data point represents signal counts per nuclear for a motor. The data bars are expressed as mean ± SEM. In B and F, statistical analysis was conducted via one-way ANOVA followed by Tukey's multiple comparisons test. In D, H and J, statistical analysis was conducted using unpaired *t*-test. *P < 0.05, **P < 0.01, ***P < 0.001, ****P < 0.0001. KD = knockdown.

We next examined the effect of TDP-43 loss on *NEAT1* expression. Knockdown of TDP-43 by two siRNAs in Neuro2a (Fig. 2A) reduced the expression of *Neat1*-total and *Neat1_2* (Fig. 2B). Considering the extractability of *Neat1_2*, retesting qRT-PCR with RNA extraction after heating treatment at 55°C led to the same result of reduced *Neat1* (Fig. S2). Image analysis with *in situ* hybridisation revealed that the expression of *Neat1_2* was reduced in TDP-43-knockdown Neuro2a compared with controls (Fig. 2C and D). Whereas, the reduction of *Neat1*-total and *Neat1_2* expressions was restored by siRNA-resistant TDP-43 (Fig. 2E and F), suggesting that *Neat1* reduction is not an off-target effect of TDP-43 knockdown. In addition, we evaluated the effects of TDP-43 knockdown in primary neurons. We knocked down TDP-43 in the primary cortical neurons of TDP-43^flox/flox^ mice^9^ via the transduction of EGFP-Cre using lentivirus (Fig. 2G) and observed a significant reduction in *Neat1* expression (Fig. 2H). Next, we performed ISH and IF on the spinal cords of TDP-43-cKO mice,^9^ in which TDP-43 was knocked out specifically in the motor neurons, as well as the control mice (Fig. 2I). Image analysis revealed that the expression of *Neat1* was reduced in TDP-43-KO motor neurons compared with that in the control motor neurons (Fig. 2J). These findings strongly suggest that *NEAT1* reduction results from the TDP-43 function loss in spinal motor neurons.

### TDP-43 binds to the *NEAT1* promoter region as a transcription factor

**Figure 3:**
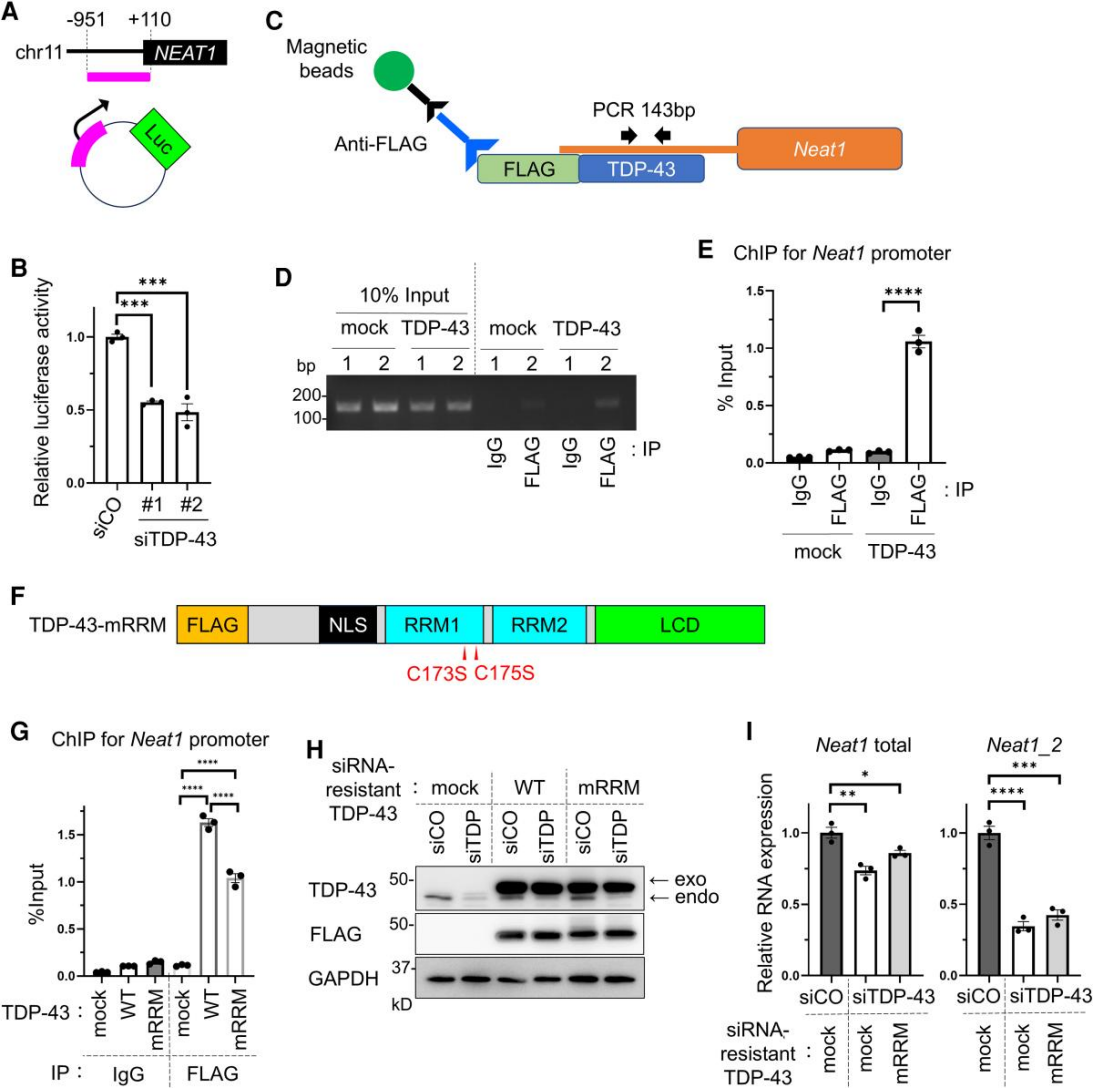
**TDP-43 regulates *NEAT1* as a transcription factor**. (**A**) Schematic illustration of the *NEAT1* promoter luciferase vector. Chr, chromosome. Luc, luciferase. (**B**) Relative luciferase activity of Neuro2a cells expressing *NEAT1* promoter luciferase and treated with the control siRNA or siTDP-43 (*n* = 3 for each group). (**C**) Schematic illustration of the chromatin immune precipitation assay. Neuro2a cells were transfected with FLAG-TDP-43 or mock. ChIP assays were conducted using anti-FLAG of mouse IgG. (**D**) Electrophoresis of the *Neat1 promoter* region (143 bp) amplified by paired primers. See supplementary Fig. 8 for uncropped gel. (**E**) Quantification of the *Neat1 promoter* region normalized with input samples via qPCR (*n* = 3 for each group). (**F**) Schematic illustration of TDP-43 mRRM, in which the RRM1 domain is modified. (**G**) ChIP assays in Neuro2a cells transfected with FLAG-TDP-43 or FLAG-TDP-43-mRRM were conducted using anti-FLAG or mouse IgG. Quantification of the *Neat1 promoter* region normalized with input samples via qPCR (*n* = 3 for each group). (**H**) Immunoblots of Neuro2a cell lysates expressing FLAG-siRNA-resistant-TDP-43, FLAG-siRNA-resistant-TDP-43-mRRM, or mock vector and treated with control siRNA or siTDP-43. In the TDP-43 immunoblot lane, the upper and lower bands indicate transfected TDP-43 and endogenous TDP-43, respectively. See supplementary Fig. 9 for uncropped blots. (**I**) Quantification of the mRNA expression level of *Neat1*-total and *Neat1_2* using cells in H (*n* = 3 for each group). In B, E, G and I, each individual data point represents the mean value for a culture well. The data bars are expressed as mean ± SEM. Statistical analysis was conducted via one-way ANOVA followed by the Tukey's multiple comparisons test. *P < 0.05, **P < 0.01, ***P < 0.001, ****P < 0.0001, ns, not significant; IP, immune precipitation.

To confirm whether TDP-43 regulates *NEAT1* at the transcriptional level, we prepared the construct (*NEAT1* promoter luciferase) that connects a luciferase downstream of the *NEAT1* promoter region (−951 to +110) (Fig. 3A).^30^ We conducted luciferase reporter assay via transfection of the *NEAT1* promoter luciferase plasmid into Neuro2a and simultaneously knocked down TDP-43 with two different siRNAs. Subsequently, we found that TDP-43 knockdown significantly reduced the activity of the *NEAT1* promoter (Fig. 3B). Next, to confirm whether TDP-43 binds to the *NEAT1* promoter region, we transfected FLAG-TDP-43 or a control vector into Neuro2a cells and conducted chromatin immunoprecipitation assay using anti-FLAG antibodies or control mouse IgG (Fig. 3C). The immunoprecipitated DNA was subjected to quantitative PCR using primers that detect the *Neat1* promoter region, and we found that TDP-43 bound to the promoter region of *Neat1* (Fig. 3D and E). These data suggest that TDP-43 acts as a transcription factor for *NEAT1*. TDP-43 is reported to have a high affinity for ssDNA with four or more TG repeats and also binds to five TT repeats.^35,36^ Although the *Neat1* promoter in the mouse genome has two TG repeats and four TT repeats, TDP-43 may bind the *Neat1* promoter through the TT repeats. As TDP-43 is known to bind DNA by its RRM domain,^37,38^ we used TDP-43-mRRM, in which RRM domain is mutated,^26,28^ to investigate whether the RRM domain is required for binding to the *Neat1* promoter (Fig. 3F). TDP-43-mRRM is expressed in the nucleus [40]. Essentially, the mutations in RRM1 significantly disrupted the binding affinity of TDP-43 for the *Neat1* promoter region (Fig. 3G). Furthermore, siRNA-resistant-TDP-43-mRRM did not restore *Neat1* expression, which was reduced by TDP-43 knockdown (Fig. 3H and I). These results suggest that TDP-43 regulates *NEAT1* at the transcriptional level via RRM1.

### *Neat1* deficiency exacerbates motor neuron degeneration and accelerates disease onset in hSOD1^G93A^ mice

We demonstrated that the loss of TDP-43 function, which is the hallmark of ALS pathogenesis, causes *NEAT1* depletion. The next research question is whether reduced *NEAT1* contributes to neurodegeneration. We knocked down *Neat1* in Neuro2a cells using siRNA and found that *Neat1* depletion significantly reduced cell viability (Fig. S3). However, it has been demonstrated that *Neat1*-KO mice exhibit normal motor performance.^39^ Therefore, we decided to use an ALS mouse model and a *Neat1*-KO mouse to investigate the role of *NEAT1* under the pathological condition of ALS. Because there is no established ALS mouse model recapitulating the TDP-43 pathology in patients with sporadic ALS, we used human SOD1^G93A^ transgenic (hSOD1^G93A^) mice. Several studies already reported lack of TDP-43 abnormalities in mutant SOD1 transgenic mice.^40,41^ Since TDP-43-cKO mice have reduced *Neat1* expression in the motor neuron without TDP-43 as described above, we refrained from using TDP-43-KO mice as ALS model mice in this experiment. FISH and IF analyses revealed that the *Neat1* expression was increased in the spinal motor neurons of 8-week-old hSOD1^G93A^ mice compared with those of control mice (Fig. S4), corresponding to motor neurons with nuclear TDP-43 in sporadic ALS cases.

**Figure 4:**
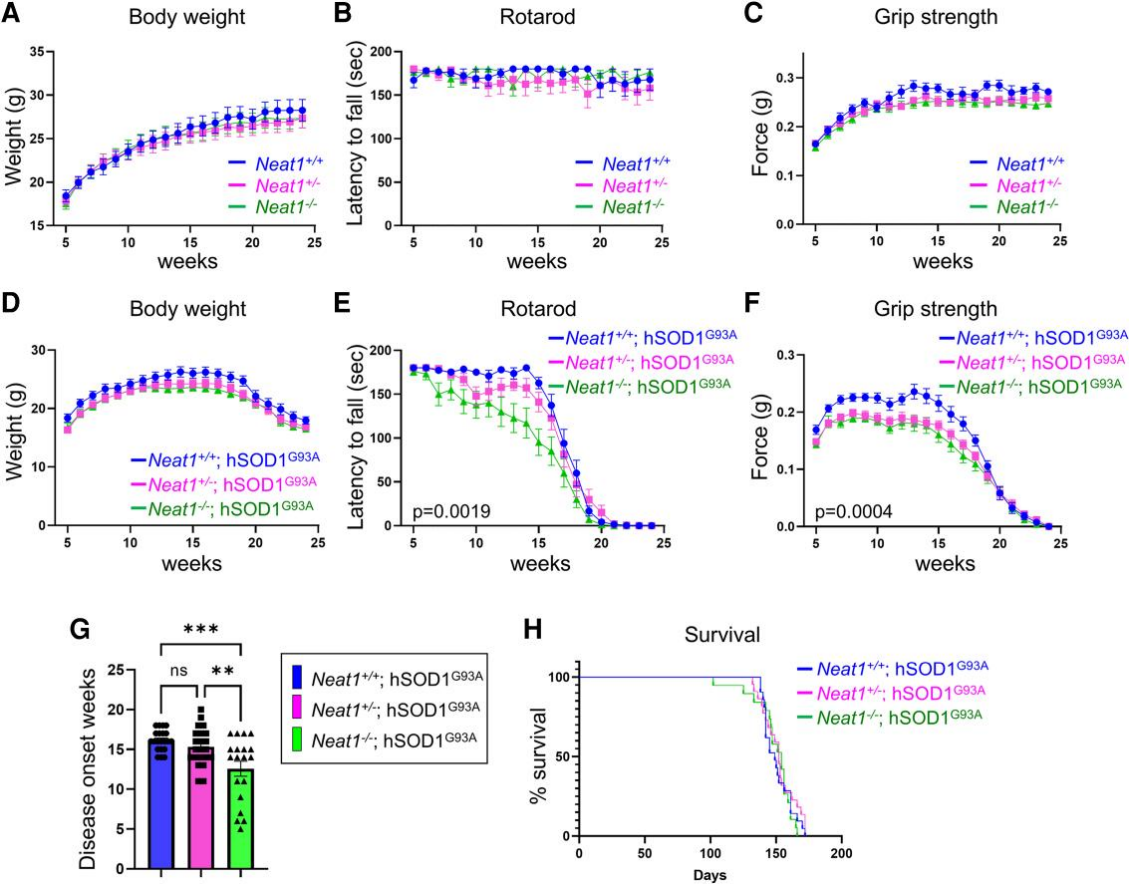
***Neat1*-KO accelerates disease onset in hSOD1^G93A^ mice**. (A–F) Behavioural assessment of each genotype of mice: *Neat1*^+/+^ (*n* = 12), *Neat1*^+/−^ (*n* = 14), *Neat1*^−/−^ (*n* = 15), *Neat1^+/+^*; hSOD1^G93A^ (*n* = 21), *Neat1^+/−^*; hSOD1^G93A^ (*n* = 22), *Neat1^−/−^*; hSOD1^G93A^ (*n* = 19). Different colours indicate different genotypes. Each individual data point represents mean of each group. Body weight (**A** and **D**), rotarod test (**B** and **E**), and grip strength (**C** and **F**) were measured every week. The data are expressed as mean ± SEM. Statistical analysis was conducted via two-way ANOVA followed by Tukey's multiple comparisons test. The results of the two-way ANOVA were *P* = 0.0019 (**E**), *P* = 0.0004 (**F**), not significant (A–D). *P < 0.05, **P < 0.01 were shown in *Neat1*-KO; hSOD1^G93A^ versus *Neat1*-WT; hSOD1^G93A^ (**E** and **F**). (**G**) Disease onset in hSOD1^G93A^-positive group. Each individual data point represents disease onset weeks for a single animal. The data bars are expressed as mean ± SEM. Statistical analysis was conducted via one-way ANOVA followed by Tukey's multiple comparisons test. **P < 0.01, ***P < 0.001, ns: not significant. (**H**) Kaplan–Meier plot of survival in the *hSOD1^G93A^*-positive group. Statistical analysis was conducted using the log-rank test. ns, not significant. (G and H; *Neat1^+/+^*; hSOD1^G93A^, *n* = 21; *Neat1^+/−^*; hSOD1^G93A^, *n* = 22; *Neat1^−/−^*; hSOD1^G93A^, *n* = 19).

To examine the effects of *NEAT1* deficiency on ALS pathology, we crossed *Neat1*-KO mice with hSOD1^G93A^ mice. First, we crossed *Neat1*^+/−^ mice^31^ with hSOD1^G93A^ mice. By crossing *Neat1*^+/−^; hSOD1^G93A^ male mice with *Neat1*^+/−^ female mice, we generated each genotype of mice (*Neat1*^+/+^, *Neat1*^+/−^, *Neat1*^−/−^, *Neat1^+/+^*; hSOD1^G93A^, *Neat1^+/−^*; hSOD1^G93A^, and *Neat1^−/−^*; hSOD1^G93A^) (Fig. 4A–F). *Neat1*^+/+^ was denoted as *Neat1*-WT and *Neat1*^−/−^ as *Neat1*-KO. Each week from 5 to 24 weeks of age, we measured the body weight and grip strength of the mice and examined their locomotor performances using the rotarod test. The hSOD1^G93A^-negative groups exhibited no difference in body weight (Fig. 4A), rotarod test (Fig. 4B), or grip strength (Fig. 4C). In the hSOD1^G93A^-positive groups, there was no difference in body weight (Fig. 4D); however, reduced motor performance was observed earlier in the *Neat1-KO*; hSOD1^G93A^ mice than in the other hSOD1^G93A^-positive genotypes in the rotarod test (Fig. 4E) and grip strength (Fig. 4F). The disease onset was ∼3.5 weeks earlier in the *Neat1*-KO; hSOD1^G93A^ than in the *Neat1*-WT; hSOD1^G93A^ mice (12.57 weeks versus 16.14 weeks) (Fig. 4G). Disease onset was defined as the last week when the mice ran for 180s in the rotarod test. No difference in survival was observed among the hSOD1^G93A^-positive groups (Fig. 4H). These data indicate that *Neat1* deficiency impairs locomotor function in mice with hSOD1^G93A^ pathology.

**Figure 5:**
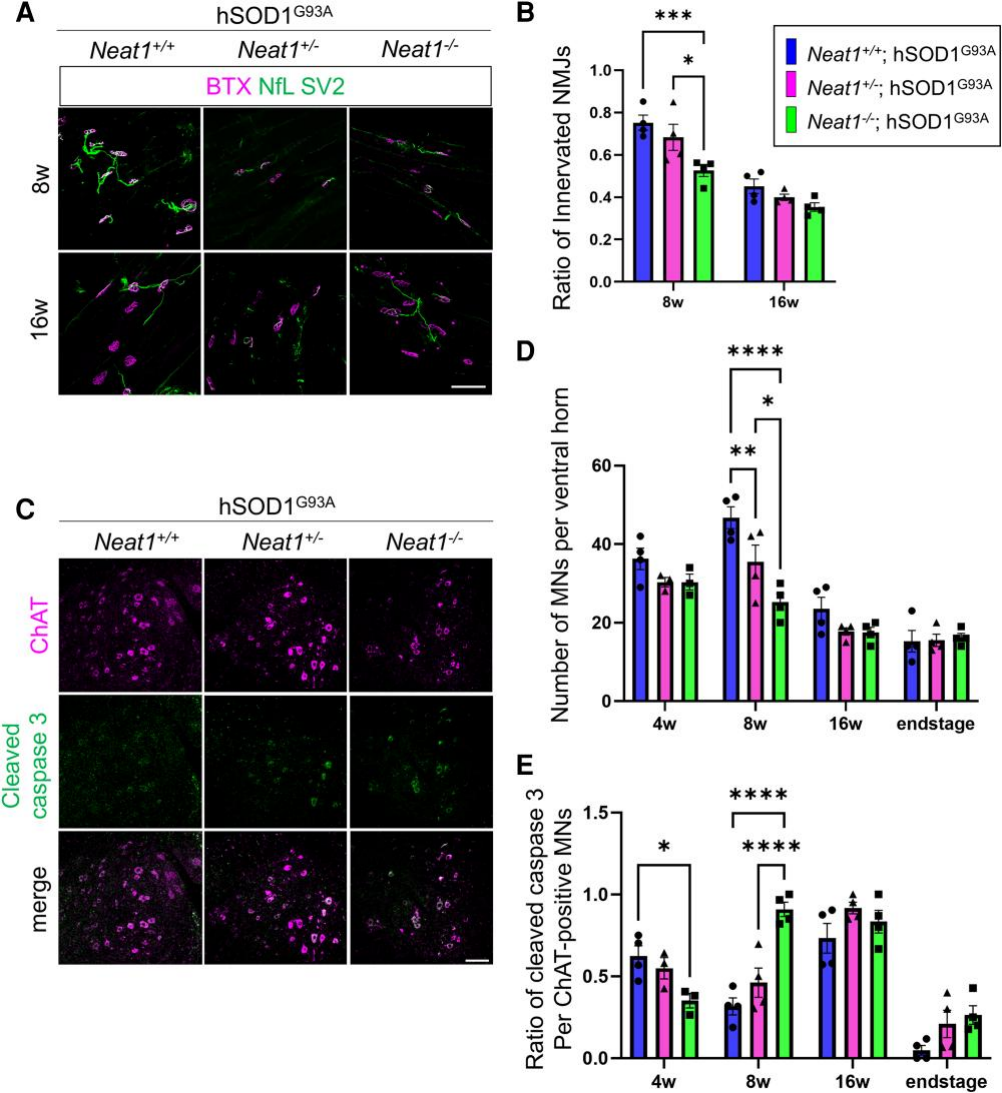
***Neat1*-KO exacerbates NMJ denervation and loss of motor neurons in hSOD1^G93A^ mice**. (**A**) Immunofluorescence images of the tibialis anterior NMJs of *Neat1*; hSOD1^G93A^ mice at 8 and 16 weeks old (α-bungarotoxin, neurofilament L and SV2). Scale bars: 100 µm. (**B**) Ratio of innervated NMJs in each genotype (*n* = 4 for each group). (**C**) Immunofluorescence images of the lumbar spinal motor neurons of *Neat1*; hSOD1^G93A^ mice at 8 weeks old (ChAT and cleaved caspase 3). Scale bars: 100 µm. (**D**) Number of motor neurons (MNs) per ventral horn in each genotype (*n* = 3–4 for group) at each week old. (**E**) Ratio of cleaved caspase-3 motoneurons per ChAT-positive motor neuron in each genotype (*n* = 3–4 for group) at each week of age. In B, D, and E, each individual data point represents the value for a single animal. The data bars are expressed as mean ± SEM. Statistical analysis was conducted via one-way ANOVA followed by Tukey's multiple comparisons test. *P < 0.05, **P < 0.01, ***P < 0.001, ****P < 0.0001. Different colours indicate different genotypes.

To examine the biological effects of *Neat1* deficiency in mice, we further analyzed the muscle and spinal cord pathology of the *Neat1*; hSOD1^G93A^ mice. To investigate neuromuscular junction (NMJ), anterior tibialis muscles were harvested from 8- to 16-week-old mice, and postsynapses were identified using α-bungarotoxin and presynapses using anti-Neurofilament L and anti-SV2 antibodies (Fig. 5A). NMJ innervation was defined as overlapping of the presynaptic labels by more than 50% of the postsynaptic labels. The results indicated that at the age of 8 weeks, the innervation rate was reduced in the *Neat1*-KO; hSOD1^G93A^ mice compared with the other groups (Fig. 5B). To analyze the motor neurons, we collected lumbar spinal cords from 4-, 8-, to 16-week-old mice as well as end-stage hSOD1^G93A^-positive mice and performed immunofluorescence staining to identify motor neurons with anti-ChAT antibody (Fig. 5C). At the age of 8 weeks, the number of motor neurons in the anterior horn of the spinal cord was significantly reduced in the *Neat1*-KO; hSOD1^G93A^ mice compared with the other groups (Fig. 5D). These data indicate that *Neat1* deficiency exacerbates NMJ denervation and motor neuron degeneration.

Because Neat1 has been demonstrated to inhibit apoptosis by blocking caspase-3 activity,^42,43^ we also counted cleaved caspase-3-positive motor neurons using an anticleaved caspase-3 antibody (Fig. 5C). We found that the ratio of cleaved caspase 3-positive motor neurons increased in the *Neat1*-KO; hSOD1^G93A^ mice at the age of 8 weeks, suggesting that apoptosis is promoted in these mice.

### *Neat1* deficiency downregulates genes involved in protein folding and accelerates the aggregation of misfolded SOD1 in the mouse spinal cord

**Figure 6:**
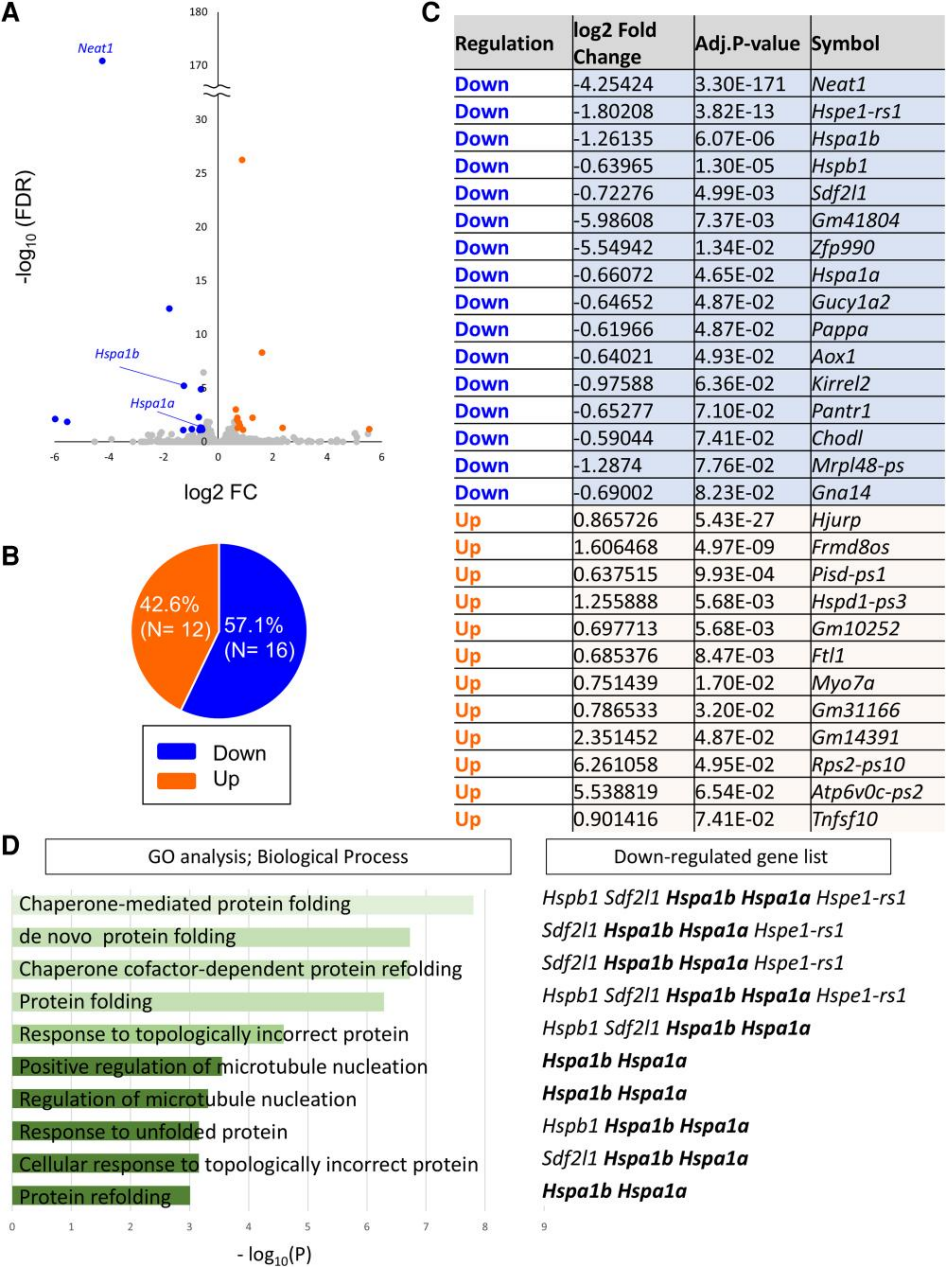
***Neat1*-KO decreases the expression of several genes involved in protein folding in hSOD1^G93A^ mice**. (**A**) Volcano plot for *Neat1*-KO; hSOD1^G93A^ mice (*n* = 4) versus *Neat1*-WT; hSOD1^G93A^ mice (*n* = 3). Up and downregulated genes with log2 fold change (log2FC) > 0.58 and adjusted *P*-value (FDR) < 0.1 are highlighted in orange and blue data points, respectively. (**B**) Proportion of up- and down-regulated genes (gene numbers are given in brackets). (**C**) List of genes showing significant changes. The direction of change as well as the log2FC and adjusted *P*-value are given. (**D**) Enrichment analysis of differential genes as analyzed using iDEP.96. GO term Biological Process and a list of genes involved in. Statistical analysis was conducted using two-tailed unpaired Student's *t*-test.

To further investigate the factors contributing to the aggravating neurodegeneration without *Neat1* in the mice, we performed RNA-sequences (RNA-seq) with RNA extracted from the spinal cords of 8-week-old *Neat1*-KO; hSOD1^G93A^ and *Neat1*-WT; hSOD1^G93A^ mice. The results indicated that the expressions of 29 genes were significantly altered: 12 and 16 genes were up- and downregulated, respectively (Fig. 6A–C). Frmd8os and Gm31166, which are close proximity to the *NEAT1* locus, were upregulated, consistent with the previous study.^39,44^ Gene ontology (GO) and pathway enrichment analysis revealed that several pathways mostly related to protein folding or refolding were significantly changed (Fig. 6D). All pathways contain *Hspa1a* and *Hspa1b* encoding encode heat-shock protein 70 (HSP70), which are significantly downregulated.

**Figure 7:**
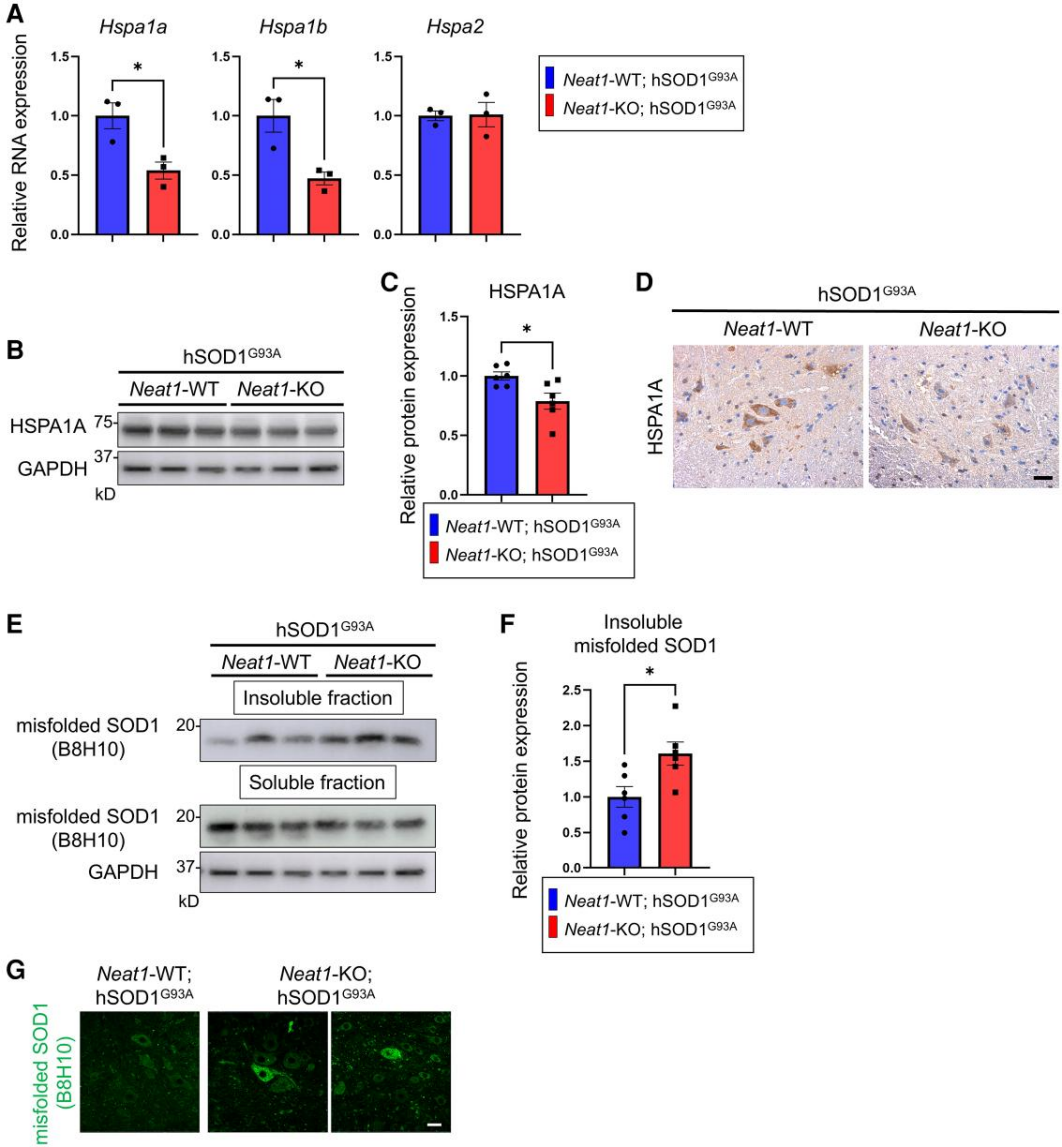
***Neat1*-KO increases the aggregation of misfolded SOD1 in hSOD1^G93A^ mice**. (**A**) Relative mRNA expression levels of *Hspa1a*, *Hspa1b*, and *Hspa2* in the cervical spinal cord (*n* = 3 for each group). *Hspa2* was measured as a negative control. (**B**) Immunoblots of cervical spinal cord lysates in *Neat1*; hSOD1^G93A^ mice. See supplementary Fig. 10 for uncropped blots. (**C**) Densitometric quantification of HSPA1A normalized with GAPDH (*n* = 6 for each group). (**D**) Immunohistochemistry of lumber spinal cord. Scale bar: 25 µm. (**E**) Immunoblots of cervical spinal cord lysates in *Neat1*; hSOD1^G93A^ mice. See supplementary Fig. 11 for uncropped blots. (**F**) Densitometric quantification of misfolded SOD1 in the insoluble fraction (*n* = 6 for each group). (**G**) Immunofluorescence image of the lumbar spinal cord (B8H10). Scale bar: 20 µm. In A, C, and F, each individual data point represents the value for a single animal. The data bars are expressed as mean ± SEM. Statistical analysis was conducted using unpaired *t*-test. *P < 0.05. Different colours indicate different genotypes. KO, knockout.

We confirmed that *Hspa1a* and *Hspa1b* mRNAs were downregulated in the spinal cords of *Neat1*-KO; hSOD1^G93A^ mice compared with *Neat1*-WT; hSOD1^G93A^ mice via qRT–PCR (Fig. 7A). Western blotting and immunostaining showed that the expression level of HSPA1A was lower in the spinal cords of *Neat1*-KO; hSOD1^G93A^ mice, consistent with the qRT–PCR (Fig. 7B–D). These results indicate that *Neat1* deficiency causes a decrease in genes related to protein folding in the spinal cords of hSOD1^G93A^ mice. We next examined changes in misfolded SOD1 expression by separating proteins from the spinal cords of mice into soluble and insoluble fractions against lysis buffer. Western blotting revealed that the amount of misfolded SOD1 protein in the insoluble fraction was increased in the spinal cords of *Neat1*-KO; hSOD1^G93A^ mice (Fig. 7E and F). Similarly, immunostaining of the mouse spinal cord showed increased expression of misfolded SOD1 in the spinal motor neurons (Fig. 7G).

## Discussion

In this study, we predicted a relationship between *NEAT1* deficiency and TDP-43 pathology from ALS postmortem spinal cords and analyzed the role of TDP-43 on *NEAT1* using cultured cells, primary neurons, and TDP-43-cKO mice, indicating that TDP-43 positively regulates *NEAT1* expression. There are several reports on the relationship between TDP-43 and *NEAT1*. In NCS-34 and SH-SY5Y cells, TDP-43 overexpression resulted in *NEAT1* upregulation.^45,46^ In MCF7 cells, TDP-43 knockdown increased *NEAT1* expression.^34^ Meanwhile, TDP-43 has been reported to upregulate *NEAT1_1* and downregulate *NEAT1_2* by binding near the 3′-end of *NEAT1_1* and regulating splicing in embryonic stem cells.^47^ These different results may be due to the different types of cells and the experimental conditions. None of the previous studies elucidated the regulatory mechanism of *NEAT1* by TDP-43, although the expression of *NEAT1* is known to be regulated by various factors in transcription, stability, and RNA processing.^48,49^ The present study demonstrated that *Neat1* expression was reduced in cultured Neuro2a cells and mouse primary cortical neurons with TDP-43 knockdown as well as in the spinal motor neurons of TDP-43-cKO mice. Moreover, promoter and ChIP assays revealed that TDP-43 is a transcriptional regulator that binds to the promoter region of *Neat1* through the RRM1 domain. These data strongly indicate that TDP-43 depletion decreases *NEAT1* expression in motor neurons; the reduced *NEAT1* expression observed in ALS motor neurons was partly caused by the reduced levels of *NEAT1* transcription due to the loss of nuclear TDP-43.

*NEAT1_2* is an essential RNA for paraspeckle formation.^19,20,50^  *NEAT1* plays a protective role in conferring stress resistance to cells by increasing its expression under stress conditions.^24,25^ It is rarely expressed in normal adult neurons; however, its expression levels are elevated in spinal motor neurons in sporadic ALS.^23,34^ In the present study, we performed immunostaining and FISH on the postmortem spinal motor neurons of patients with sporadic ALS and confirmed that, as previously reported, *NEAT1* is upregulated when nuclear TDP-43 is preserved but downregulated when nuclear TDP-43 is lost. Therefore, it is important to determine whether reduced *NEAT1* expression affects neurodegeneration in ALS patients.

*Neat1*-KO mice exhibited no abnormalities in motor function, possibly because they were not subjected to stress.^39^ Thus, to examine the effects of reduced *NEAT1* under the condition of ALS, we crossed *Neat1*-KO mice with hSOD1^G93A^ mice. As previously reported, *Neat1*-KO alone did not cause abnormal motor function in the mice. However, the *Neat1*-KO; hSOD1^G93A^ mice exhibited accelerated disease onset and intensified motor dysfunction compared with the *Neat1*-WT; hSOD1^G93A^ mice. The *Neat1*-KO heterozygous hSOD1^G93A^ mice exhibited a significantly weaker grip power and slightly worse performance in the rotarod test than the *Neat1*-WT; hSOD1^G93A^ mice. Grip analysis quantifies only limb grip strength, whereas rotarod assesses whole-body muscle strength and other motor balance. Therefore, grip strength appears to be more vulnerable to disease-related decline than other motor functions in Neat1-KO heterozygous mice. On the other hand, there is no difference in survival between *Neat1*-KO and *Neat1*-WT mice. *Neat1*-KO; hSOD1^G93A^ mice exhibited exacerbated NMJ denervation and loss of motor neurons early in disease progression at 8 weeks of age, compare with *Neat1*-WT; hSOD1^G93A^ mice, whereas the differences became less prominent at 16 weeks of age or endstage. This suggests that the effect of *Neat1*-KO in hSOD1^G93A^ mice is most prominent early in the disease progression, while the phenotype is strongly influenced by the pathology of hSOD1^G93A^ later in the disease progression. RNA-seq analysis showed that genes related to protein folding, including *Hspa1a* and *Hspa1b*, were downregulated in the *Neat1*-KO; hSOD1^G93A^ mice. Previous reports of transcriptome analysis of the cerebral cortex of *Neat1*-KO mice showed conspicuous changes in genes related to synapses and RNA processing, but no significant changes in protein folding-related genes.^39^  *Hspa1a* and *Hspa1b* are chaperone proteins that belong to the HSP70 family and protect cells against stress.^51,52^ HSP70 is also known to interact with elongated peptides and partially folded proteins, thereby preventing protein aggregation.^53,54^ In the present study, the *Neat1*-KO; hSOD1^G93A^ mice exhibited loss of spinal motor neurons and decreased innervated NMJs compared with the *Neat1*-WT; hSOD1^G93A^ mice. Moreover, protein solubility analysis revealed increased misfolded SOD1 protein aggregation. The delayed disease onset^55^ and suppressed muscle denervation^56^ of SOD1 mice overexpressing HSP70 suggest that the loss of Neat1 in hSOD1^G93A^ mice causes a decrease in HSP70, which promotes SOD1 aggregation and exacerbates neurodegeneration. In addition, we found that *Neat1* depletion increased cleaved caspase-3 expression in hSOD1^G93A^ mice. Caspase-3 is reportedly activated in the spinal cords of SOD1 transgenic mice.^57-59^ HSP70 blocks the binding of caspase-9 to the apoptosome complex, thereby suppressing caspase-3 activation and inhibiting apoptosis.^60^ Furthermore, *NEAT1*, particularly *NEAT1_2*, is known to inhibit apoptosis by promoting cell cycle and inhibiting caspase-3 activation.^42,43^ The reduction in HSP70, as well as *Neat1* depletion, may further activate caspase-3 in the motor neurons of hSOD1^G93A^ mice. The results of the current and previous studies together indicate that *Neat1*-KO has the potential to exacerbate the original SOD1 pathology. As previously discussed, *Neat1* was upregulated in the motor neurons of hSOD1^G93A^ mice and neurons with nuclear TDP-43 in sporadic ALS cases. The loss of TDP-43 function potentially enhances ALS neurodegeneration by losing the protective effect of *NEAT1*.

In conclusion, loss of nuclear TDP-43 is correlated with decreased *NEAT1* in ALS motor neurons, and TDP-43 positively regulates *NEAT1* by acting as a transcription factor. *NEAT1* deficiency exacerbates neurodegeneration in the ALS pathology by promoting aggregation of pathological proteins. Furthermore, loss of TDP-43 exacerbates protein aggregation, and such an aggregation, in turn, has the potential to accelerate the loss of TDP-43 function. This negative feedback can be a potential therapeutic target for ALS.

## Supplementary Material

fcaf261_Supplementary_Data

## Data Availability

The data that support the findings of this study are available from the corresponding author, upon reasonable request. Full-size and uncropped blots/gels are available within Supplementary material (Figs. S5–S11). RNA-sequencing data in this paper is available in the NCBI Gene Expression Omnibus (GEO) database with accession number GSE279899 [https://www.ncbi.nlm.nih.gov/geo/query/acc.cgi?acc=GSE279899].
