# Supplementary material for: Downregulation of NEAT1 due to loss of TDP-43 function exacerbates motor neuron degeneration in amyotrophic lateral sclerosis
Source: Brain Commun. 2025 Jul 2;7(4):fcaf261. doi: 10.1093/braincomms/fcaf261 (PMC12256815; doi:10.1093/braincomms/fcaf261)
Supplement: fcaf261_Supplementary_Data [file fcaf261_supplementary_data.docx]

**Downregulation of *NEAT1* due to loss of TDP-43 function exacerbates motor neuron degeneration in amyotrophic lateral sclerosis**

Yu Kawakami, Yohei Iguchi, Jiayi Li, Yoshinobu Amakusa, Takashi Yoshimura, Ryo Chikuchi, Satoshi Yokoi, Madoka Iida, Yuichi Riku, Yasushi Iwasaki, Tetsuro Hirose, Shinichi Nakagawa, Masahisa Katsuno

**Supplemental Figures 1-11**


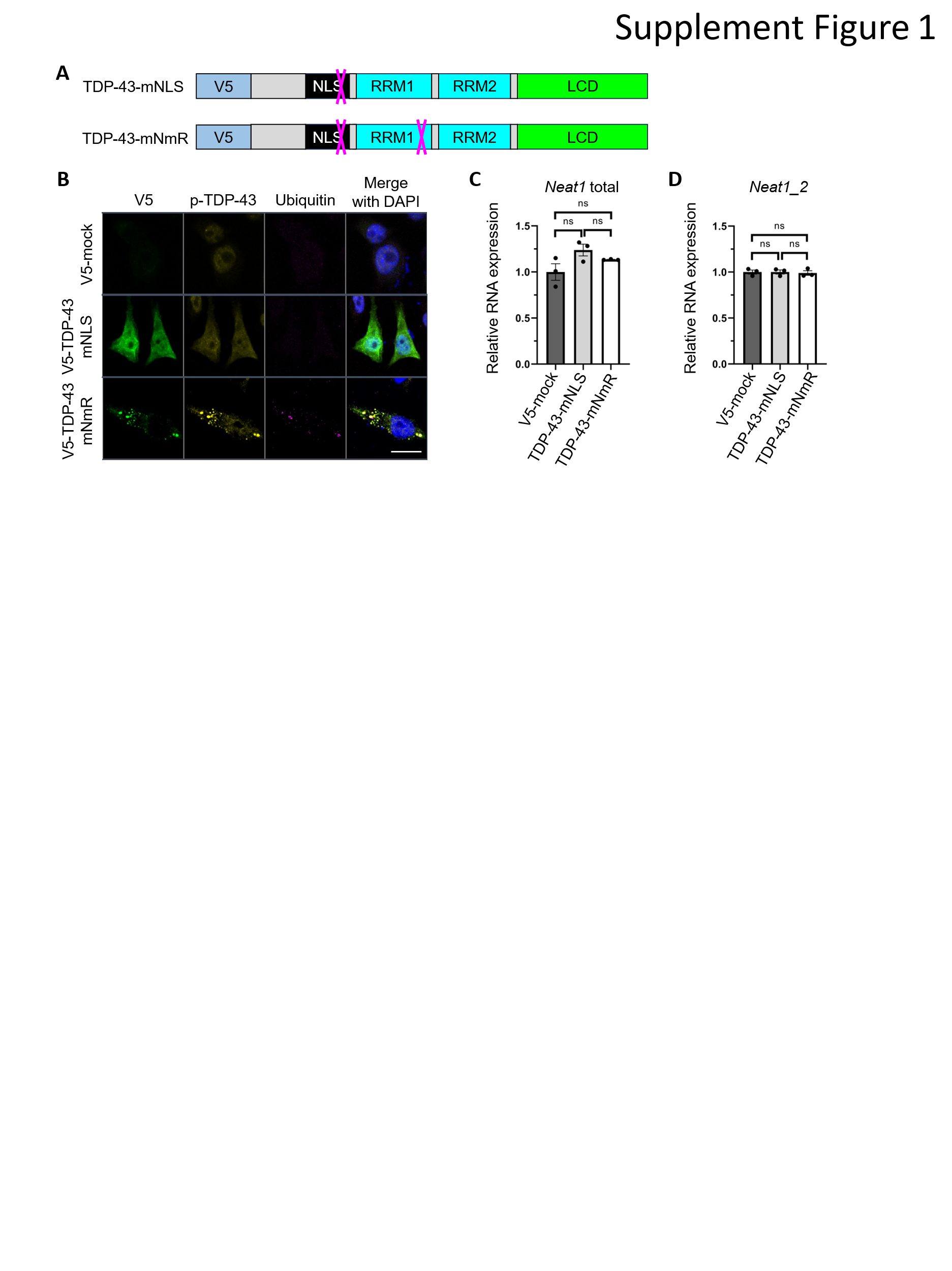


**Supplemental Figure 1. Cytoplasmic TDP-43 does not affect *Neat1* expression**

(A) Schematic illustrations of the cytoplasmic localized TDP-43 mutation, TDP-43-mNLS, in which NLS is modified, and the aggregation-prone TDP-43 mutation, TDP-43-mNmR, in which NLS and RRM1 are modified. (B) Immunofluorescence images of Neuro2a cells expressing V5-mock, V5-TDP-43-mNLS, or V5-TDP-43-mNmR (V5, green; p-TDP-43, yellow; ubiquitin, magenta; DAPI, blue). Scale bar: 10 µm. (C, D) Quantification of the mRNA expression levels of *Neat1*-total (C) and *Neat1_2* (D) using cells in B (n = 3 for each group). In C and D, each individual data point represents the average of three measurement for a culture well. The data bars are expressed as mean ± SEM. Statistical analysis was conducted via one-way ANOVA followed by Tukey’s multiple comparisons test. ns: not significant.


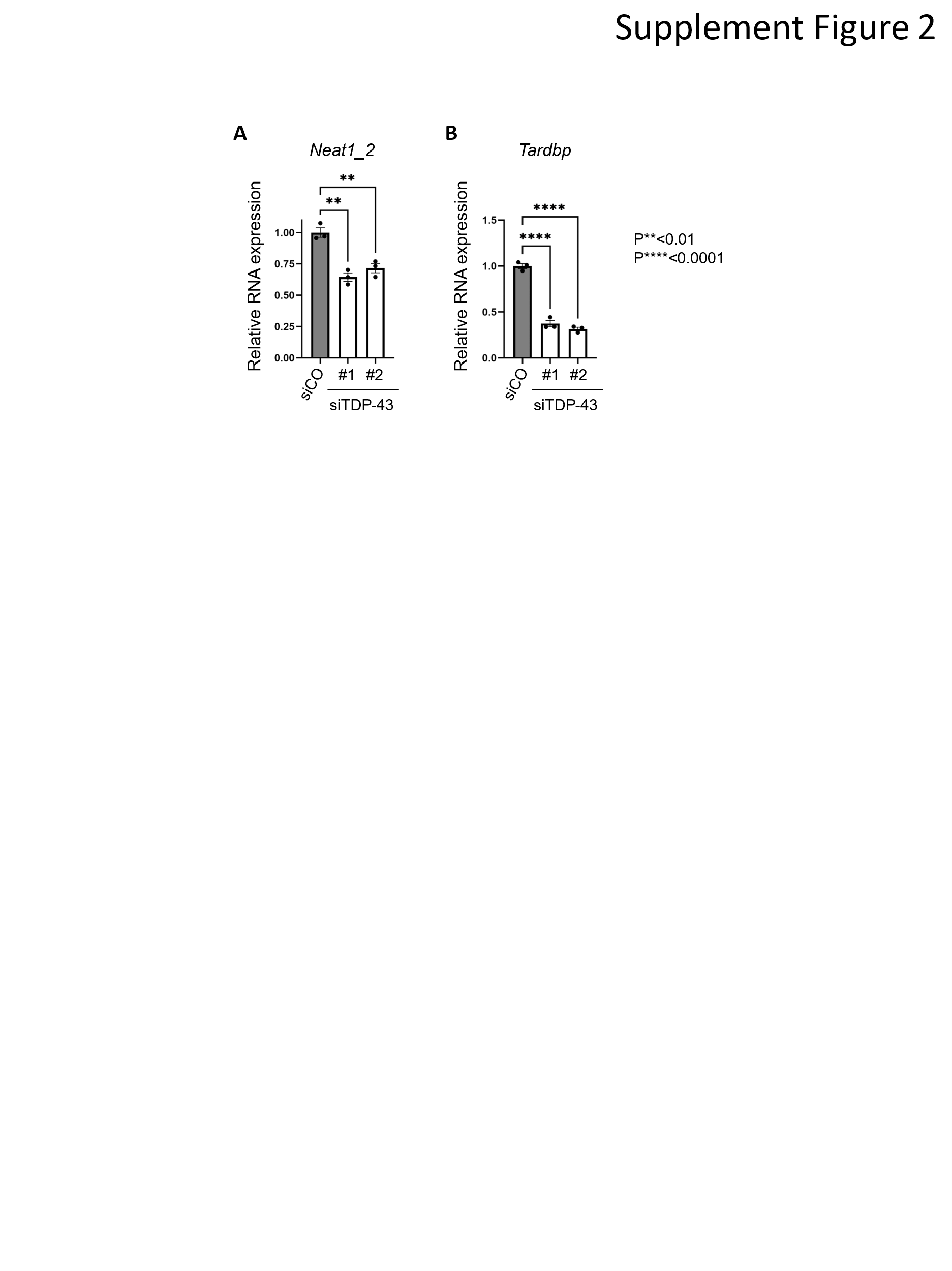


**Supplemental Figure 2. Even under heating at 55°C, knockdown of TDP-43 induces *Neat1_2* reduction**

(A and B) Prior to total RNA extraction, cell lysate in QIAzol reagent was heated at 55°C for 10 min. Quantification of the mRNA expression levels of *Neat1*_2 (A) and *Tardbp* (B) using Neuro2a treated with siTDP-43 (n = 3 for each group). In A and B, each individual data point represents the average of three measurement for a culture well. The data bars are expressed as mean ± SEM. Statistical analysis was conducted via one-way ANOVA followed by Tukey’s multiple comparisons test. ***P*< 0.01, *****P*< 0.0001.


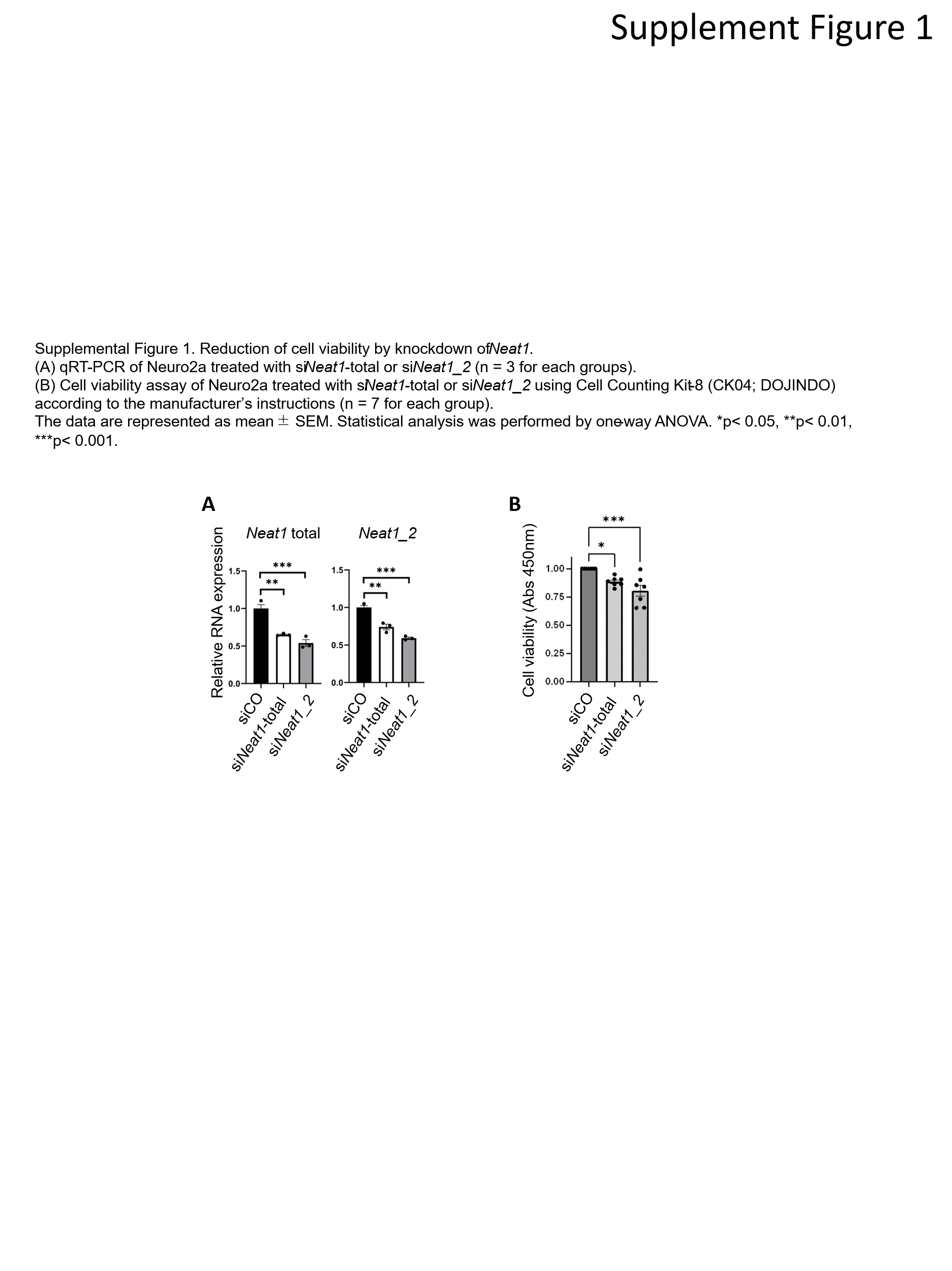


**Supplemental Figure 3. Reduction of cell viability by knockdown of *Neat1***

(A) Quantitative RT-PCR of Neuro2a treated with si*Neat1*-total or si*Neat1_2* (n = 3 for each group). (B) Cell viability assay of Neuro2a treated with si*Neat1*-total or si*Neat1_2* using Cell Counting Kit-8 (CK04; DOJINDO) according to the manufacturer’s instructions (n = 7 for each group). Each individual data point represents the average of three measurement for a single well. The data bars are represented as mean ± SEM. Statistical analysis was performed by one-way ANOVA. **P*< 0.05, ***P*< 0.01, ****P*< 0.001.


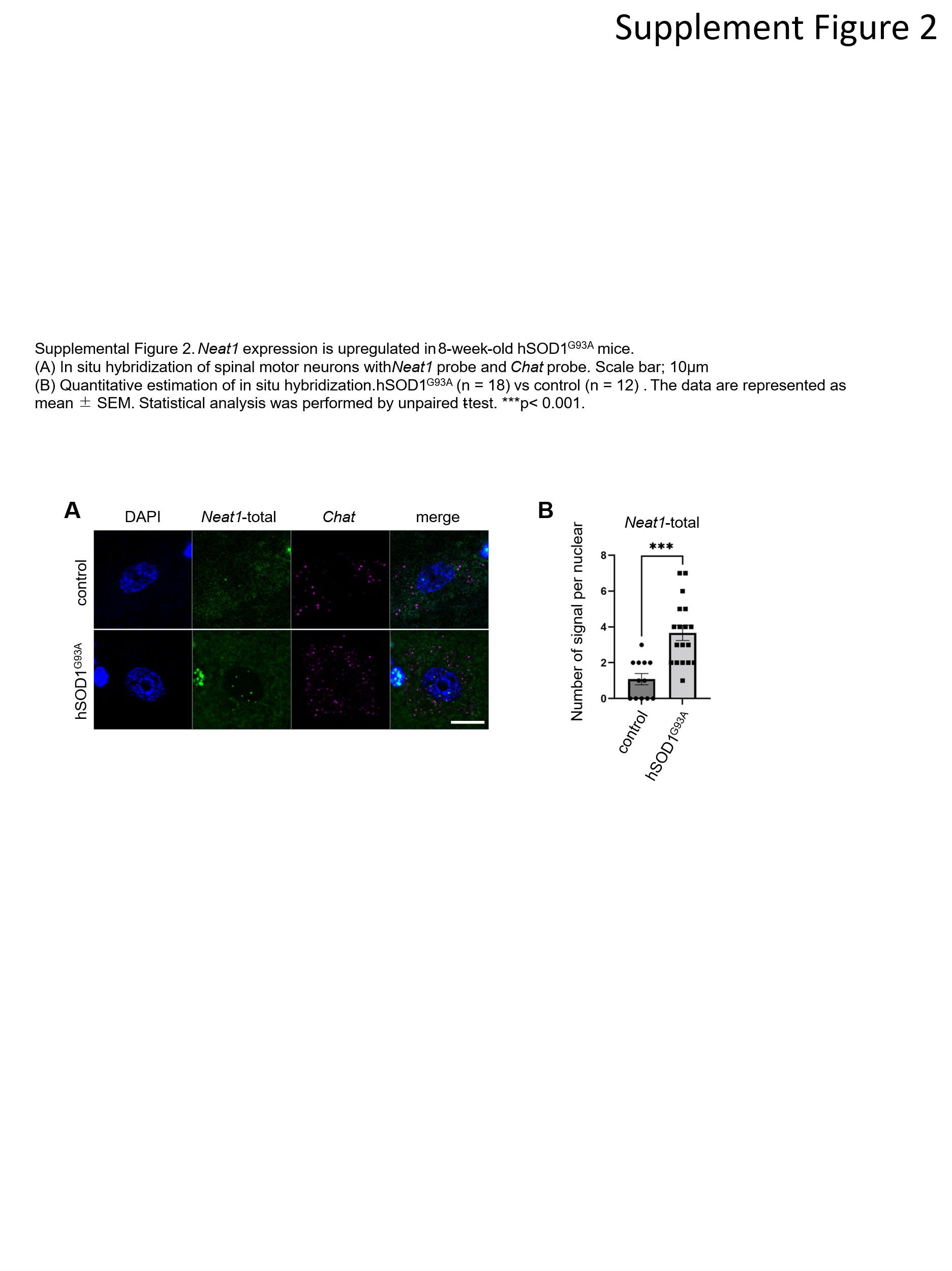


**Supplemental Figure 4. *Neat1* expression is upregulated in 8-week-old hSOD1^G93A^ mice**

(A) In situ hybridization of spinal motor neurons with *Neat1* probe and *Chat* probe. Scale bar; 10µm. (B) Quantitative estimation of in situ hybridization. Each individual data point represents signal counts per nuclear for a single motor neuron. hSOD1^G93A^ (n = 18) vs control (n = 12). The data bars are represented as mean ± SEM. Statistical analysis was performed by unpaired t-test. ****P*< 0.001.


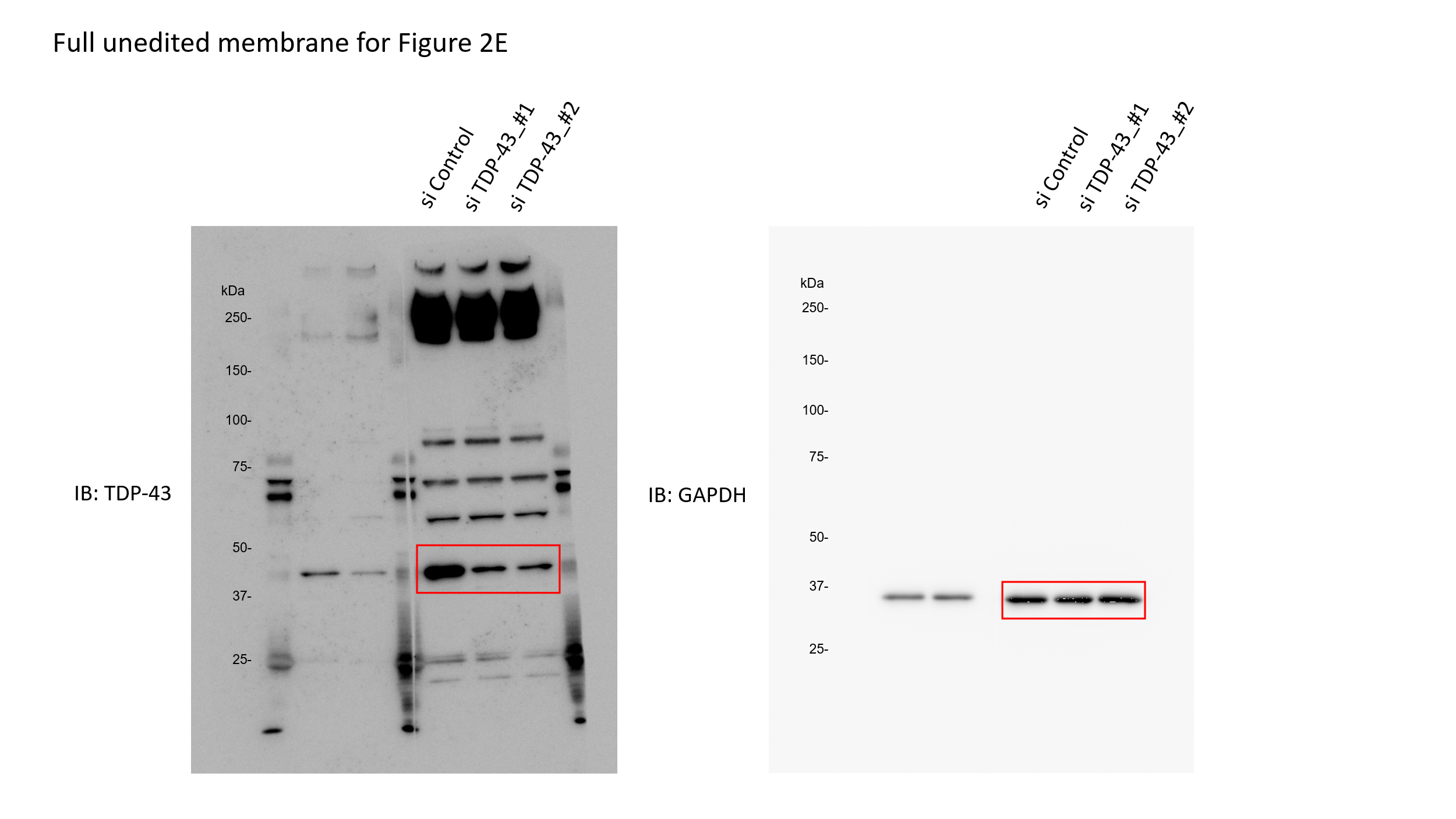


**Supplemental Figure 5. Uncropped membrane corresponding to Figure 2A**


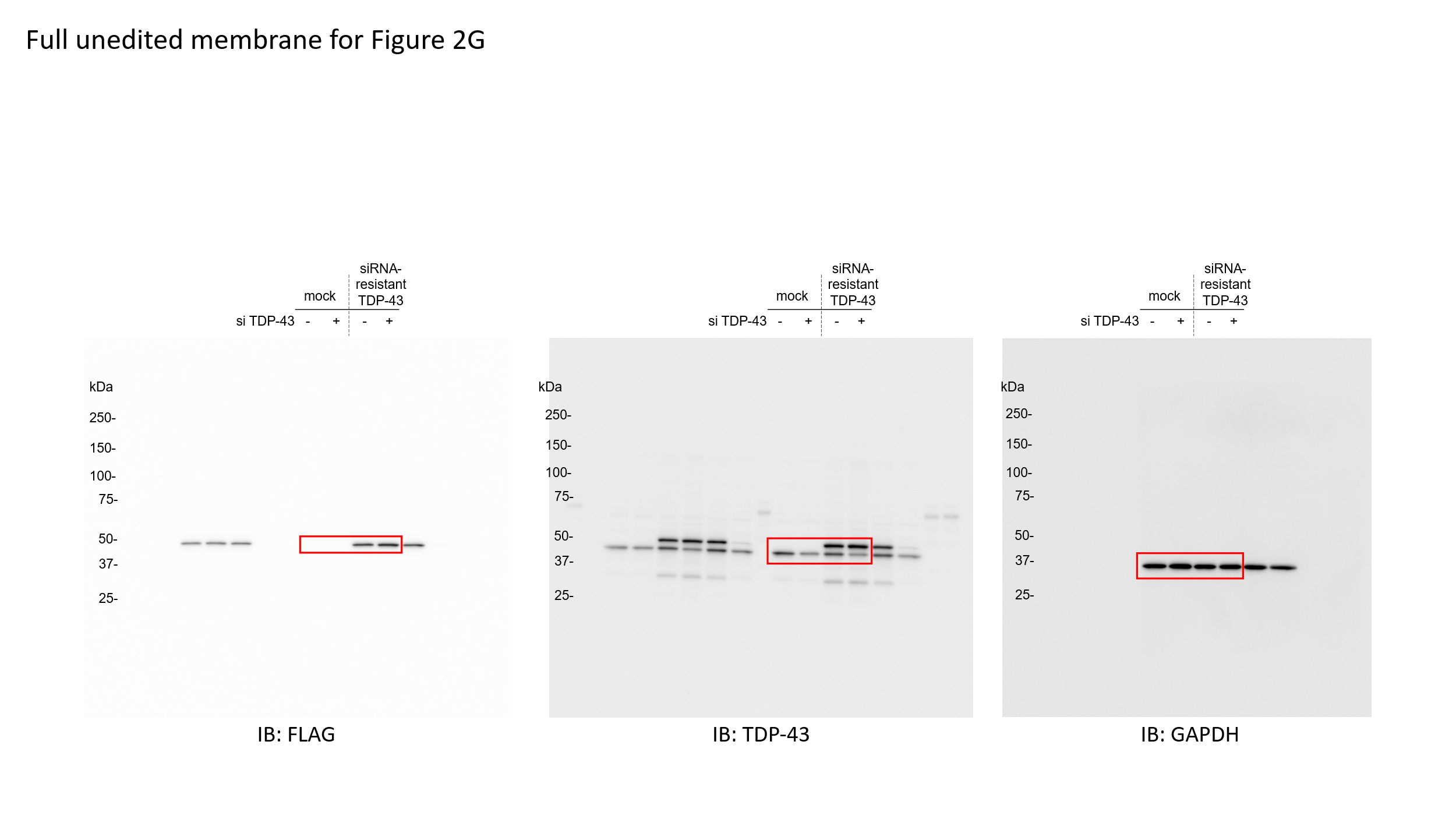


**Supplemental Figure 6. Uncropped membrane corresponding to Figure 2E**


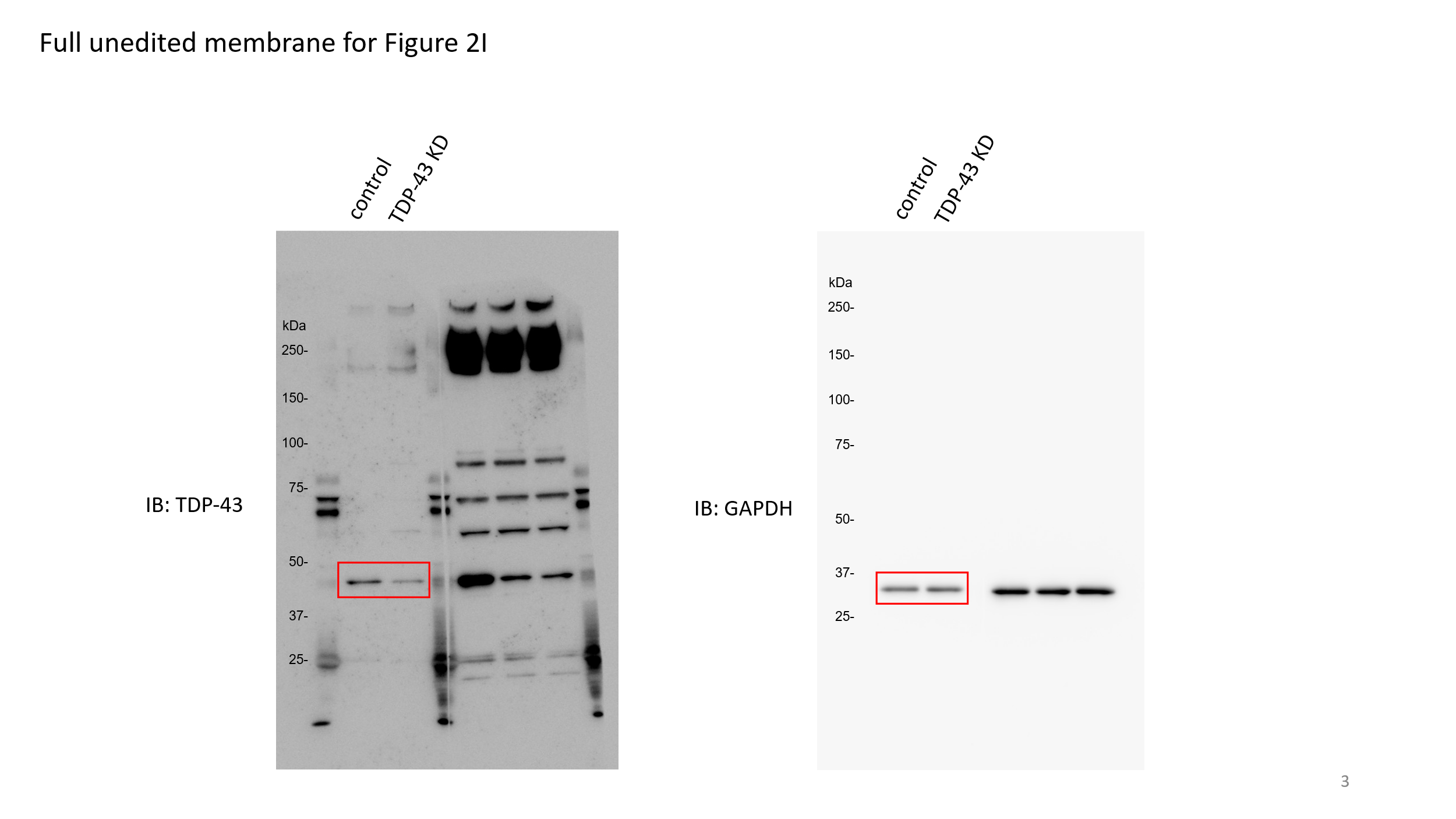


**Supplemental Figure 7. Uncropped membrane corresponding to Figure 2G**


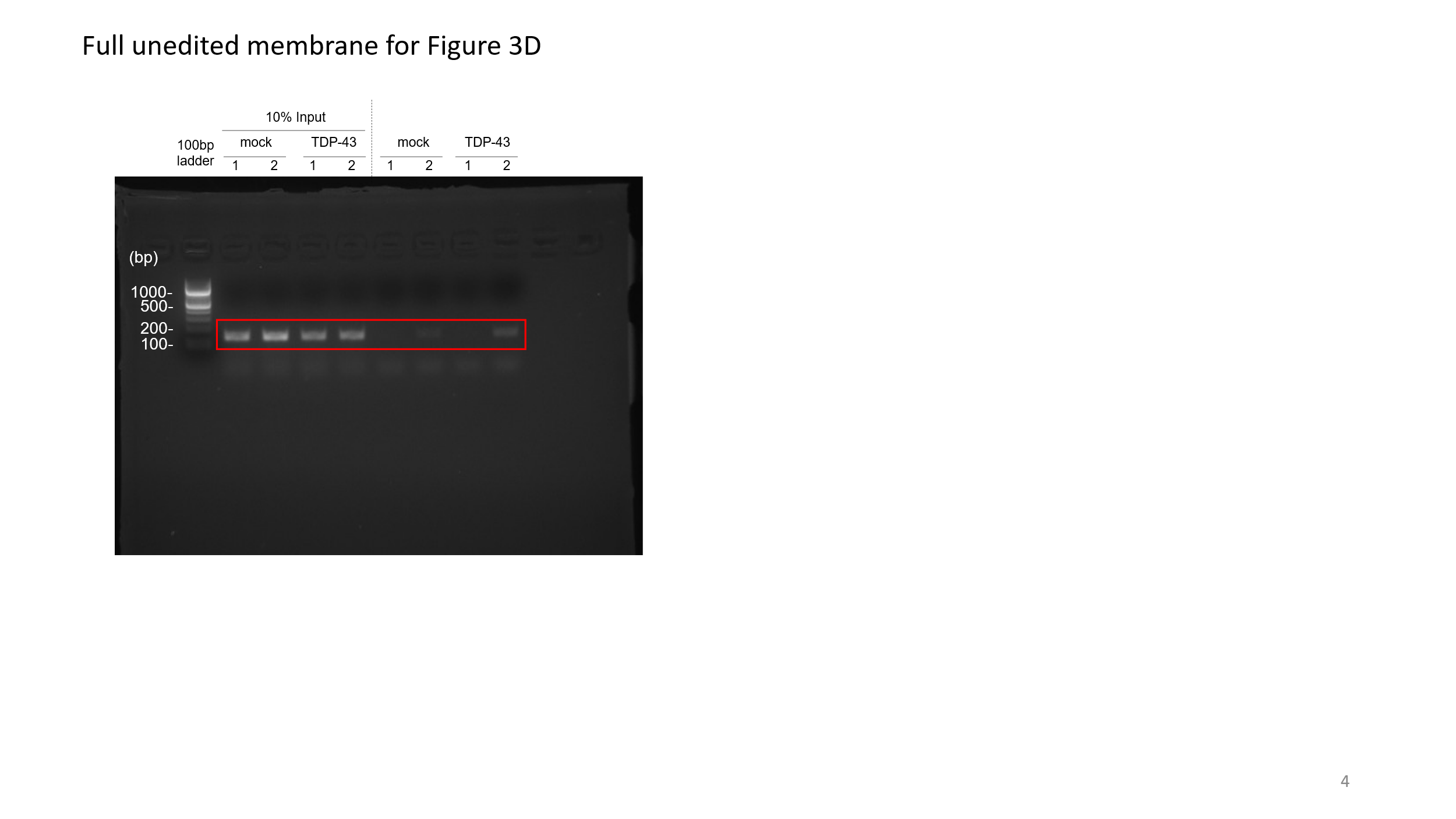


**Supplemental Figure 8. Uncropped membrane corresponding to Figure 3D**


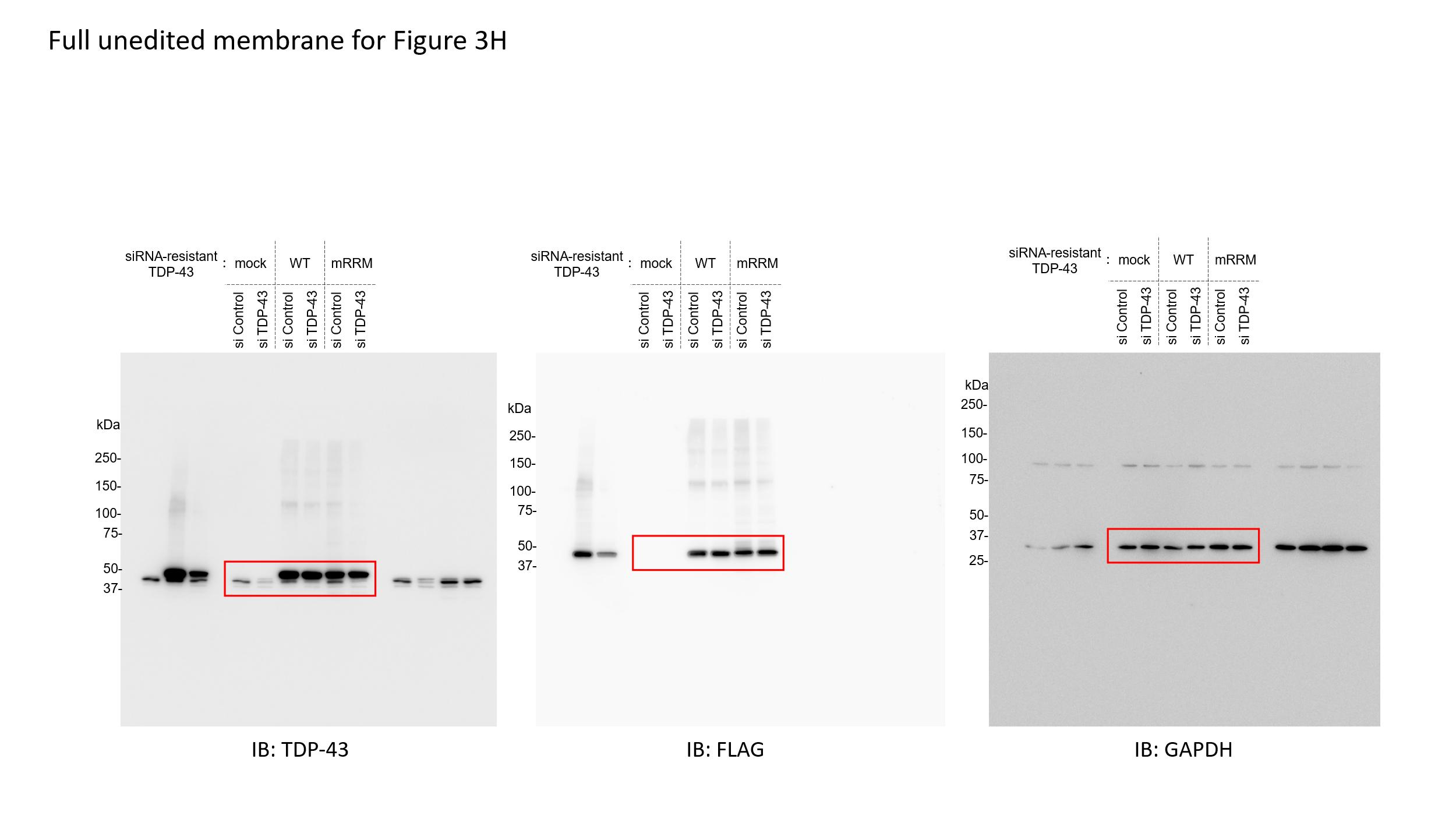


**Supplemental Figure 9. Uncropped membrane corresponding to Figure 3H**


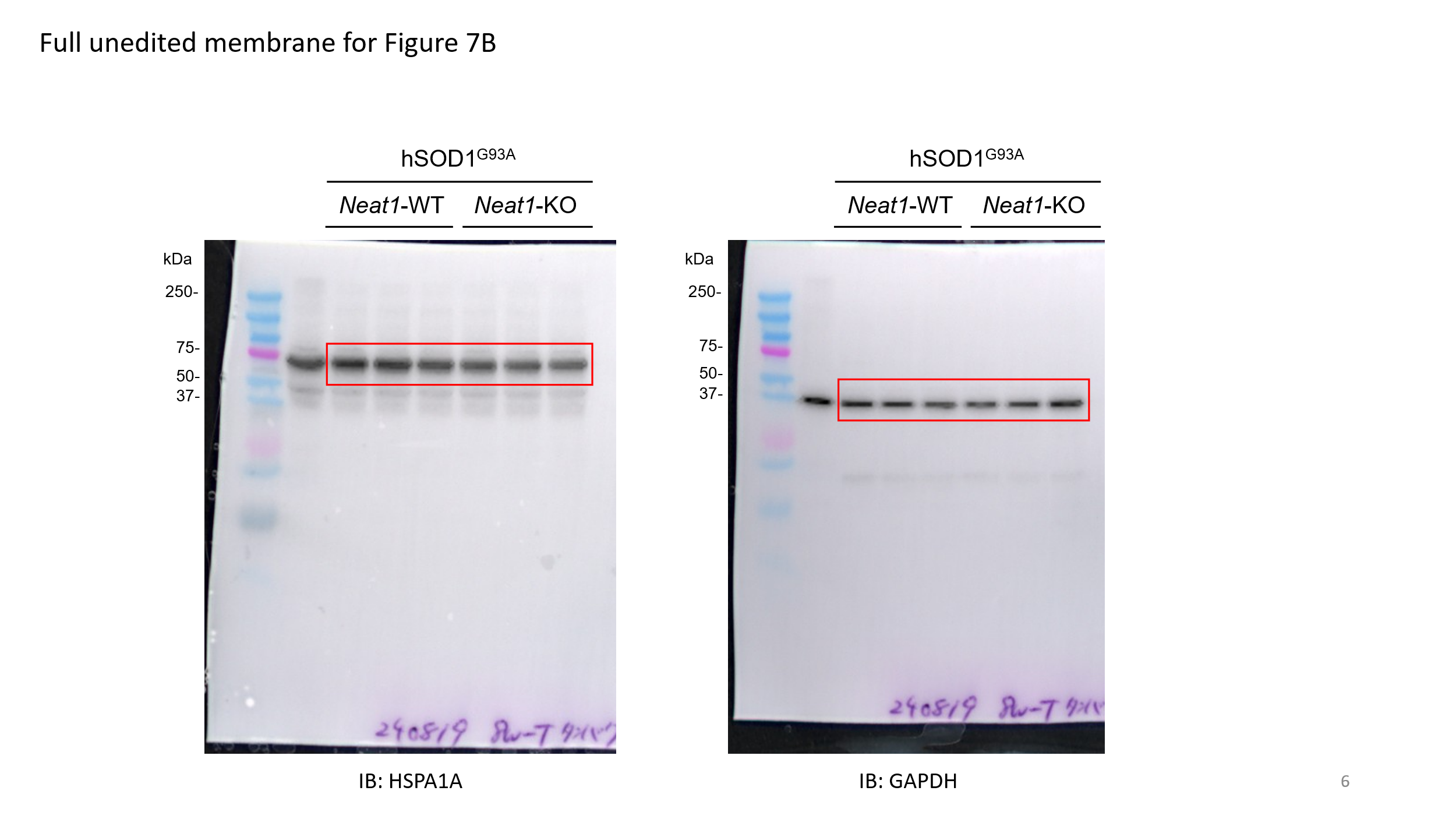


**Supplemental Figure 10. Uncropped membrane corresponding to Figure 7B**


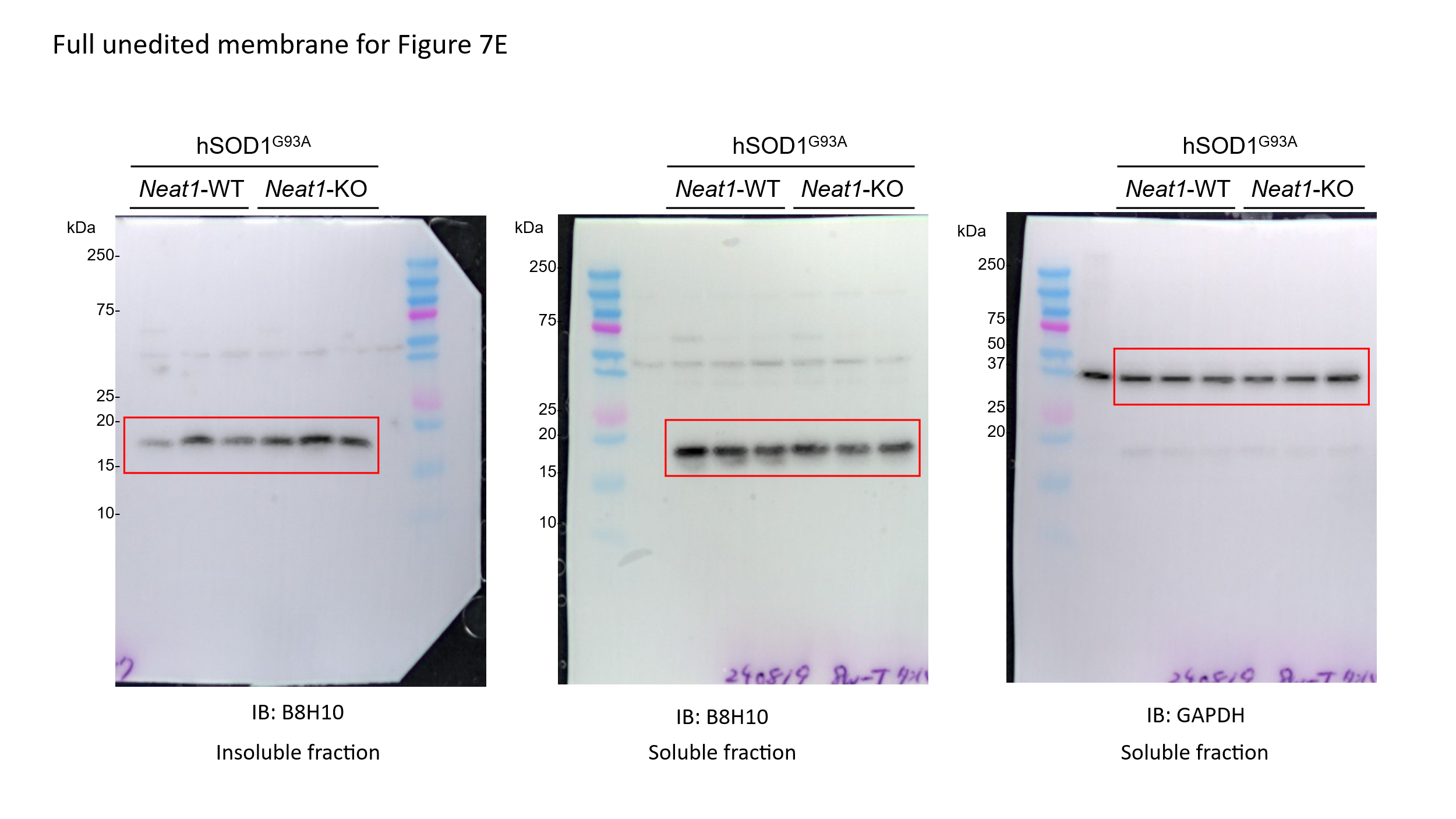


**Supplemental Figure 11. Uncropped membrane corresponding to Figure 7E**
